# Deep Learning Approaches for Quantifying Ventilation Defects in Hyperpolarized Gas Magnetic Resonance Imaging of the Lung: A Review

**DOI:** 10.3390/bioengineering10121349

**Published:** 2023-11-23

**Authors:** Ramtin Babaeipour, Alexei Ouriadov, Matthew S. Fox

**Affiliations:** 1 School of Biomedical Engineering, Faculty of Engineering, The University of Western Ontario, London, ON N6A 3K7, Canada; rbabaeip@uwo.ca; 2 Department of Physics and Astronomy, The University of Western Ontario, London, ON N6A 3K7, Canada; mfox28@uwo.ca; 3 Lawson Health Research Institute, London, ON N6C 2R5, Canada

**Keywords:** deep learning, Magnetic Resonance Imaging (MRI), hyperpolarized gas MRI, segmentation, ventilation defect, chronic obstructive pulmonary disease (COPD), lung imaging

## Abstract

This paper provides an in-depth overview of Deep Neural Networks and their application in the segmentation and analysis of lung Magnetic Resonance Imaging (MRI) scans, specifically focusing on hyperpolarized gas MRI and the quantification of lung ventilation defects. An in-depth understanding of Deep Neural Networks is presented, laying the groundwork for the exploration of their use in hyperpolarized gas MRI and the quantification of lung ventilation defects. Five distinct studies are examined, each leveraging unique deep learning architectures and data augmentation techniques to optimize model performance. These studies encompass a range of approaches, including the use of 3D Convolutional Neural Networks, cascaded U-Net models, Generative Adversarial Networks, and nnU-net for hyperpolarized gas MRI segmentation. The findings highlight the potential of deep learning methods in the segmentation and analysis of lung MRI scans, emphasizing the need for consensus on lung ventilation segmentation methods.

## 1. Introduction

In 2016, lung diseases such as chronic obstructive pulmonary disease (COPD), lower respiratory tract infections, cancer, and tuberculosis were included in the top ten leading causes of death worldwide [1]. As a result, imaging of the lungs plays a pivotal role in initial diagnosis. At present, Computed Tomography (CT) is considered the gold standard for lung imaging [2,3,4]; however, Magnetic Resonance Imaging (MRI) presents a promising alternative for obtaining radiation-free images of the lungs [5]. 

MRI is a non-invasive and non-ionizing imaging technique, meaning that it does not require surgical intervention or the use of harmful ionizing radiation to create detailed images of the internal structures of the body. To be more specific, MRI eliminates the need for any exploratory surgery or the insertion of probes into the body [6]. Additionally, MRI relies on strong magnetic fields and radio waves to generate images, rather than harmful ionizing radiation [7]. These two features of MRI make it an ideal imaging modality for studying lung function and assessing pulmonary diseases. 

When it comes to imaging the lung, there are certain limitations of conventional MRI. Conventional MRI focuses on detecting hydrogen nuclei, specifically protons (^1^H), to generate images of internal structures. Due to the low proton density of the lung and susceptibility effects on relaxation at the air/tissue interfaces within the alveoli, conventional MRI struggles to provide high-quality images [8,9,10].

Conversely, hyperpolarized agents have been introduced for use in MR imaging, offering a means to address and overcome certain limitations associated with conventional MRI techniques. Hyperpolarized agents such as helium-3 (^3^He) or xenon-129 (^129^Xe), are noble gases that can have their nuclear magnetic resonance (NMR) signal significantly increased after undergoing a hyperpolarization process. This leads to dramatic improvement in the signal-to-noise ratio (SNR) compared with conventional MRI [11].

Ventilation defects are characterized by areas in the lungs where airflow is impaired or blocked, leading to reduced or even absent gas exchange [12,13]. These defects can manifest in several forms such as obstructions in the small airways, mucus accumulation, broncho-constriction, or tissue damage [14]. Consequently, the distribution of ventilation within the lungs becomes heterogeneous [15]. Given the benefits conferred by hyperpolarized agents, ^3^He and ^129^Xe MRI have proven to be excellent tools for identifying ventilation irregularities [16]. They are particularly effective in populations such as elderly individuals who have never smoked [17], as well as patients diagnosed with COPD [18,19,20,21,22,23,24,25], asthma [26,27,28], Cystic Fibrosis (CF) [29,30,31], radiation-induced lung injury [32,33], and those who have received lung transplants [34,35].

The current golden standard [36] for calculating the VDP involves a semi-automated method. While the semi-automated method for estimating the ventilation defect percent (VDP) is effective, it is not without its shortcomings. A significant limitation lies in the time-intensive nature of the process. As an illustration, the segmentation and calculation procedure for an 80-slice lung MRI can consume approximately 45 min. This time requirement can be a significant bottleneck in a clinical setting where time efficiency is critical.

In recent years, the advancement of deep learning has ushered in a new era for medical imaging, including image segmentation [37,38,39]. DL-based models, harnessing the power of artificial intelligence, have demonstrated exceptional prowess in the automatic segmentation of medical images, revolutionizing this complex task. These models have proven to be highly efficient and accurate, outperforming traditional methods and offering a transformative approach to medical image analysis. DL methods have shown great success in many areas of medical image analysis [40]. These methods are both efficient and accurate. They have been used for tasks such as identifying brain tumors [41], separating the lungs in CT images [42], aiding in breast cancer radiotherapy [43], and tracking lung changes in COPD [44]. Deep learning has shown promising results in hyperpolarized gas MRI. In a research article [45], deep learning was utilized to improve image reconstruction quality. A deep cascade of residual dense networks (DC-RDNs) was crafted to reconstruct high-quality DW images from substantially undersampled k-space data. The study utilized hyperpolarized ^129^Xe lung ventilation images from 101 participants to create synthetic DW-MRI data, which then trained the DC-RDN. The performance of the DC-RDN was subsequently assessed using both retrospectively and prospectively undersampled multiple b-value ^129^Xe MRI datasets, demonstrating its effectiveness in enhancing DW-MRI for lung morphometry. Another study also introduced PhysVENeT [46], a 3D multi-channel convolutional neural network that synthesizes 3D ventilation surrogates from MRI scans. This approach, which incorporates physiologically informed ventilation mapping and structural imaging, showed superior performance over conventional mapping and other DL methods, offering an accurate reflection of ventilation defects with minimal overfitting. Several studies have also used DL techniques for the segmentation of ventilated lungs [47,48,49].

This manuscript aims to present a review of DL-based automatic segmentation techniques as applied to hyperpolarized MRI of the lung. We will explore the ways these advanced techniques are enhancing our ability to identify and quantify ventilation defects, thereby offering potential improvements in the diagnosis and management of lung diseases.

## 2. The Current Golden Standard

“Hyperpolarized ^3^He Magnetic Resonance Functional Imaging Semiautomated Segmentation” [36] presents a comprehensive study on the application of manual and semi-automated volume measurements in the context of hyperpolarized ^3^He MRI of the lung. The study’s primary goal was to explore the efficiency and accuracy of semiautomated segmentation methods in the analysis of ventilation defects in lung diseases such as asthma, COPD, and CF. This study involved a total of 15 subjects, with each group consisting of 5 subjects with asthma, COPD, and CF, respectively. The demographic characteristics of these subjects were meticulously recorded and analyzed, providing a robust foundation for the study’s findings. 

The study utilized multistep segmentation software developed in MATLAB R2007b. The segmentation of ^3^He MR images was initiated using a hierarchical K-means clustering algorithm [50], a histogram-based method that partitions observations into clusters based on the nearest mean. 

Additionally, ^1^H MR images were segmented using a Seeded Region-Growing Algorithm (SRGA) [51]. This pixel-based method begins with a seed point and expands the region by selecting neighboring pixels with similar properties. The process continues until no further growth of the region is possible.

The segmented ^1^H MR images were subsequently registered, as referenced in [52] to segmented^3^He MRimages. This process distinguished ventilation defects from the background, resulting in the creation of a ^3^He voxel cluster map. 

The study demonstrates that the semiautomated volume measurement method is as reliable as manual methods for patients with asthma, COPD, and CF. The strong correlation between the two methods and minimal bias, as shown with Bland–Altman analysis, supports the semiautomated approach’s accuracy in quantifying ventilation defects in hyperpolarized ^3^He MRI of the lung.

**Figure 1:**
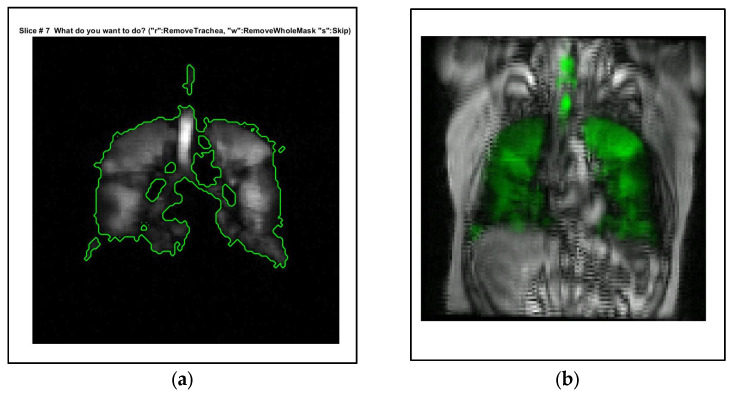
Illustration of the semi-automated segmentation method and its limitations. (**a**) Significant manual intervention is required in the process, particularly in the removal of the trachea from the hyperpolarized gas MRI segmentation. Description of what is contained in the first panel. (**b**) A visual representation of the registration process using landmarks, demonstrating the intricate steps involved in the segmentation method. The green color indicates the distribution of hyperpolarized gas. Screenshots taken using MATLAB R2021b.

However, the semiautomated method has limitations. It requires about 45 min per patient, which is still substantial, especially in clinical settings. Additionally, it requires significant manual intervention, such as excluding the trachea from the hyperpolarized gas MRI segmentation, adding to the overall time and effort. Figure 1 visualizes the semi-automated process and its limitations.

These limitations highlight the need for a more efficient, fully automated process. DL-based segmentation methods show promise in automating complex tasks like image segmentation, reducing the need for manual intervention.

## 3. Overview of Deep Neural Networks 

In this part of our review, we examine the fundamental principles and structures of DL. We initiate our discussion by providing a thorough outline of key types of Deep Neural Networks. Following this, we focus on the diverse layers that make up deep networks, detailing their specific roles and functions. We then emphasize the importance of evaluation metrics, which serve as crucial tools for gauging the effectiveness of these models. Finally, we transition to a discussion on the commonly used architectures in medical image segmentation, highlighting their prominence in the field.

### 3.1. Deep Neural Network Architectures

#### 3.1.1. Convolutional Neural Networks (CNNs)

Convolutional Neural Networks (CNNs) have established themselves as some of the most impactful and commonly used architectures within the DL networks, particularly for tasks related to computer vision. Waibel et al. [53] further developed CNNs by introducing shared weights across temporal receptive fields and using backpropagation training for phoneme recognition. LeCun et al. [54] also contributed to the field by designing a CNN architecture specifically for the task of document recognition.

Convolution is a critical mathematical operation within the structure of CNNs. It operates linearly, taking input data and multiplying it by specified weights. This operation is executed with the assistance of a filter or mask, which is essentially a two-dimensional weight array and is generally smaller than the input data. The procedure entails conducting an element-wise multiplication, or dot product, between the filter and an equivalent-sized patch of the input. This results in a single output value. This process is systematically replicated across the whole input image, creating a two-dimensional output array known as a feature map. A notable aspect of this operation is the consistent application of the same filter across the entire image, allowing the algorithm to identify a particular feature regardless of its position in the image. This key feature is referred to as translation invariance.

A key strength of CNNs is their capability to self-learn filters, eliminating the necessity for manual filter creation. By leveraging the power of backpropagation, an essential training technique, the network adjusts the weights of the filters based on the error it calculates during the training phase. This advantage provides a more dynamic and adaptive approach to identifying relevant features within the data.

**Figure 2:**
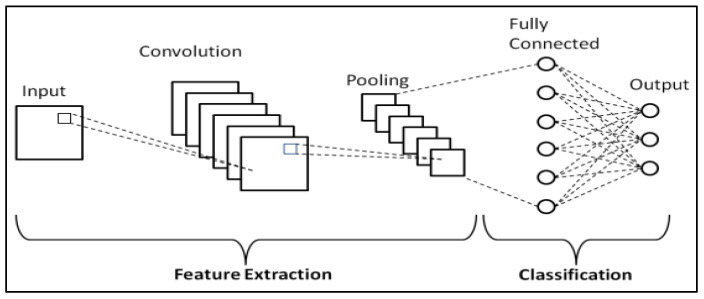
Schematic representation of CNNs [55].

The learning process occurs across various layers, each responsible for identifying distinct types of features within the data. The layers closer to the input are typically tasked with learning low-level features such as lines and edges, serving as the building blocks for more complex pattern recognition. As we move deeper into the network, the layers progressively learn to recognize more complex, higher-order features like shapes and object parts. This hierarchical learning structure allows the model to gradually construct a more intricate understanding of the input data, from simple, foundational elements to complex, composite features. A representation of the CNN general workflow is provided in Figure 2.

#### 3.1.2. Recurrent Neural Networks (RNNs)

Recurrent Neural Networks (RNNs) [56] represent another class of DL architectures that have shown significant promise in tasks involving sequential data. Each neuron in a layer of an RNN receives input not only from the previous layer but also from its previous time step, enabling the model to exhibit temporal dynamic behavior and recognize patterns across time. This makes them particularly adept for tasks such as natural language processing, speech recognition, machine translation, music generation, and sentiment classification, where the order of the input data carries significant information. Key advantages of RNNs include their ability to process inputs of any length and maintain compact model size, regardless of the larger inputs. However, RNNs do face challenges, including higher computational costs, difficulty in accessing distant past information, and issues related to the vanishing or exploding gradient problem.

**Figure 3:**
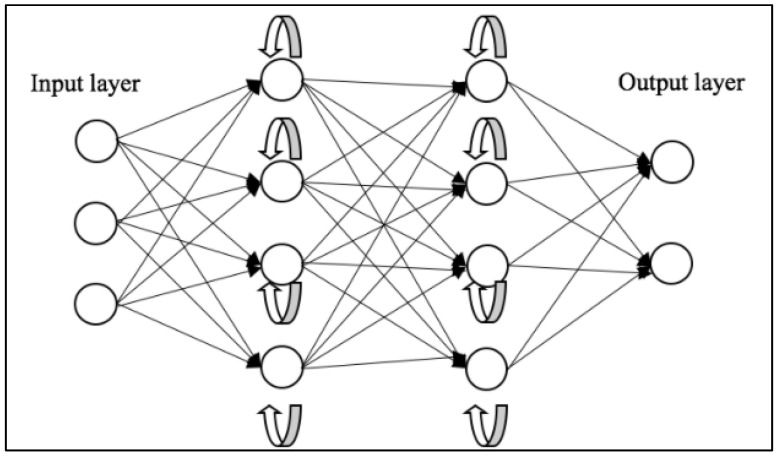
Schematic representation of RNNs [59].

Vanishing and exploding gradients are common issues encountered during the training of neural networks, particularly those with deep architectures like RNNs. Both problems can make it difficult for an RNN to learn and maintain long-term dependencies in the data. To address this, Long Short-Term Memory (LSTM) [57] networks and Gated Recurrent Unit (GRU) [58] were introduced. A depiction of the general workflow of RNNs can be seen in Figure 3.

#### 3.1.3. Encoder–Decoder Network Models

Encoder–Decoder models are a class of DL models that have shown significant results in a variety of tasks. These models consist of two main components: an encoder model that is responsible for compressing data into a compact latent representation, and a decoder model that reconstructs the original data from this latent representation. The overall goal of these models is to learn a representation of the data that can capture its structure. A notable application of encoder–decoder models can be found in medical imaging. The study titled “Low-Dose CT With a Residual Encoder-Decoder Convolutional Neural Network” by Hu Chen and colleagues (2017) [60] showcases a model that integrates an autoencoder, deconvolution network, and shortcut connections to form a residual encoder–decoder convolutional neural network (RED-CNN), specifically designed for low-dose CT imaging. The model was trained on patch-based data and demonstrated competitive performance in terms of noise suppression, structural preservation, and lesion detection.

#### 3.1.4. Generative Adversarial Networks (GANs)

Generative Adversarial Networks (GANs) are a groundbreaking and potent methodology used in unsupervised and semi-supervised learning [61]. GANs, a relatively recent innovation, draw inspiration from noise-contrastive estimation and harness implicit modeling of high-dimensional data distributions. The structure of GANs is bifurcated into two neural networks: a generative network, tasked with learning the patterns within a database to synthesize plausible new data, and a discriminative network, which categorizes samples as either original or generated data. These two networks participate in a competitive interplay, where the loss of one network equates to the gain of the other. The fundamental principle of GANs is to motivate the generator to produce a data distribution that closely mirrors that of real data, with the generative networks being indirectly trained with a dynamically updated discriminative network.

**Figure 4:**
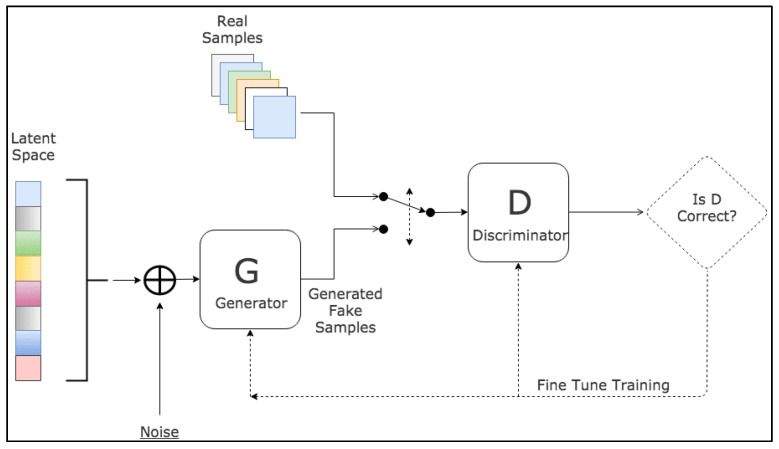
Schematic representation of GANs [66].

Since their introduction, GANs have been extensively applied across a wide range of tasks in computer vision, including image segmentation. Various GAN architectures have been developed and deployed, such as fully connected GANs [62], Convolutional GANs [63], Conditional GANs [64], Wasserstein-GAN [65], GANs with inference models, and Adversarial autoencoders. The adaptability and effectiveness of GANs have led to their incorporation in numerous studies and projects, showcasing their potential to enhance accuracy and enable semi-weakly supervised semantic segmentation. As the field continues to progress, GANs are anticipated to assume an increasingly pivotal role in the advancement of deep learning and computer vision. A visual representation of the general workflow of GANs is illustrated in Figure 4.

### 3.2. Deep Neural Network Layers

#### 3.2.1. Activation Layer

In deep learning models, the activation layer is essential as it determines the necessary information for accurate predictions. Typically found after the convolutional layers in CNNs, these layers function on feature maps. The activation function within these layers decides if a neuron’s input should be activated based on the input’s significance for the model’s prediction accuracy. These functions must be efficient due to the fact that they will be applied across millions of neurons. Moreover, in CNNs, backpropagation requires these functions to be non-linear, demanding additional computational efficiency. Over the years, several activation functions such as sigmoid [67], softmax [68], Rectified Linear Units (ReLU) [69], Leaky ReLU [70], and the recently introduced Swish [71] have been developed.

The sigmoid function,

(1) $$\sigma (x)=\frac{1}{1+e^{-x}}$$

which ranges from 0 to 1, is ideal for models predicting probabilities. The SoftMax function

(2) $$\sigma (\vec{z}{)}_{i}=\frac{e^{z_{i}}}{\sum_{j=1}^{K}\;\;e^{z_{j}}}$$

is a more general sigmoid function commonly used for multi-class classification. However, these functions can be computationally intensive and may face the issue of vanishing gradients.

ReLU

(3) ReLU (x)=max(0,x)

is a widely used activation function because of its simplicity and ability to handle the vanishing gradient problem. However, it can experience the “dying ReLU” problem as it outputs zero for all negative inputs, inhibiting backpropagation.

Leaky ReLU,

(4) $$Leaky\;ReLU\left(x\right)=max(0.01\times x,x)$$

an improved version of ReLU, addresses this problem but may introduce some inconsistencies for negative input predictions. Both Leaky ReLU and ReLU can still encounter the exploding gradient problem with larger input values.

Parametric ReLUs (PReLUs)

(5) PReLU (x)=max(0,x)+α×min(0,x)

extend this concept by transforming the leakage coefficient into a parameter that is learned concurrently with the other parameters of the neural network. Here, α is a learnable parameter. This innovative approach allows the network to self-adjust the coefficient based on the specific requirements of the data, thereby enhancing the adaptability and performance of the model.

Lastly, the Swish function,

(6) $$Swish\;(x)=\frac{x}{1+e^{-x}}$$

a further improvement of ReLU developed by Google researchers, maintains computational efficiency and can sometimes outperform ReLU. 

#### 3.2.2. Pooling and Batch Normalization

Pooling layers are a crucial component of CNNs. Pooling layers are generally placed between consecutive convolutional layers within a CNN architecture. Their main role is to diminish the spatial dimensions of the representation, which in turn reduces the number of parameters and computational load in the network. This controls overfitting and makes the network invariant to small translations. Multiple types of pooling operations exist, with Max pooling and average pooling being the most commonly utilized.

Max pooling is the most common type of pooling, and it involves defining a spatial neighborhood (for example, a 2 × 2 window) and taking the largest element from the rectified feature map within that window. Instead of taking the average element value, we take the maximum element. This highlights the most present feature in the local region of the feature map. Max pooling has been favored in practice due to its performance in experiments and its ability to preserve the presence of the feature, no matter how small. Average pooling is a process that computes the average value for each patch on the feature map. This means that each max pooling operation (over a 2 × 2 window) is replaced with an unweighted average over the same window. This was often used traditionally but has recently been largely replaced with max pooling, which performs better in practice.

Pooling layers provide a form of translation invariant representation in the feature map, which can be beneficial in image classification tasks where objects may appear in different locations of the input image1. Pooling layers contribute to making the representation approximately invariant to minor translations of the input. This translation invariance is particularly beneficial when the presence of a feature is more significant than its precise location. Batch normalization [72] is a strategy used for efficiently training deep neural networks. It involves standardizing the inputs to a specific layer across each mini-batch, thereby stabilizing the learning process and markedly decreasing the number of epochs required to train deep networks. This technique was introduced to tackle the issue of internal covariate shift, a phenomenon where the distribution of inputs for each layer shifts during the training process due to changes in the parameters of preceding layers. This can slow down the training process and make it difficult to train models with saturating nonlinearities. It has been shown to have a dramatic effect on the optimization performance of neural networks. Moreover, batch normalization can also act as a form of regularization, effectively reducing the generalization error, similar to how activation regularization functions.

#### 3.2.3. Optimizers

Optimizers play a crucial role in deep learning, as they are responsible for updating the weight parameters and minimizing the loss function. They are the driving force behind the training of deep learning models, and their efficiency can significantly impact the performance of these models. In the context of deep learning, several optimizers have been developed to improve the performance of models.

Stochastic Gradient Descent (SGD) [73] is one of the most basic optimization algorithms in deep learning. It uses a fixed learning rate for all parameters throughout the entire training process, and it is known for its fast convergence ability.

The Adaptive Gradient Algorithm (Adagrad) [74] is an optimizer that uses different learning rates for each parameter in the model. It adapts the learning rate according to the frequency of each parameter’s update, which can be particularly beneficial for dealing with sparse data and large-scale problems.

Root Mean Square Propagation (RMSProp) [75] is an optimization algorithm designed to address the diminishing learning rates in Adagrad. RMSProp uses an exponentially decaying average to discard history from the extreme past, allowing it to converge rapidly after finding a convex bowl.

Adadelta [76] builds upon Adagrad’s framework, aiming to mitigate its steeply declining learning rate. Unlike Adagrad, which accumulates all historical squared gradients, Adadelta limits the scope of these gradients to a constant window, denoted as ‘w’. This is achieved by setting the state as a progressively diminishing average of past squared gradients. Adam (Adaptive Moment Estimation) [77] is another popular optimizer that computes adaptive learning rates for different parameters. It stores an exponentially decaying average of past gradients similar to momentum and RMSProp. This characteristic renders it particularly effective for problems with large datasets or a high number of parameters.

DL optimizers are essential for effectively training models and achieving high performance. The choice of optimizer can significantly impact the model’s ability to learn from the data and generalize well to unseen data.

#### 3.2.4. Fully Connected Layers

Fully connected layers, also known as dense layers, play a crucial role in the architecture of neural networks. These layers are characterized by their interconnectivity, where each neuron in a layer is connected to all neurons in the previous layer. This dense interconnection allows for the integration of learned features from prior layers, thus enabling the network to learn more complex representations. Fully connected layers are typically used toward the end of a neural network, following several convolutional or recurrent layers that are responsible for feature extraction. The role of the fully connected layers is to take these extracted features and combine them in various ways to solve the problem the network is designed to address, such as classification or segmentation [78].

#### 3.2.5. Dropout Layer

Dropout layers [79] are a powerful and intuitive regularization technique. Dropout operates on the principle of randomly omitting units and their connections within the neural network during the training phase. By doing so, it hinders excessive co-adaptation among units, resulting in a network with enhanced generalization capabilities. During training, dropout effectively trains a large ensemble of thinned networks with shared weights, where each thinned network gets trained very rarely, if at all. This effectiveness stems from the fact that with each instance of training, a newly ‘thinned’ version of the network is generated and subjected to training. The thinned networks are derived from the original network by removing non-output units independently with a certain probability during the training phase. Dropout has demonstrated a notable ability to curb overfitting, yielding substantial enhancements compared with other regularization techniques.

### 3.3. Evaluation Metrics

Assessment metrics are vital for evaluating the effectiveness of semantic segmentation models in deep learning. These metrics provide quantitative measures that reflect the accuracy and effectiveness of the model in segmenting images. They are essential in comparing different models, tuning model parameters, and improving model performance. In the medical imaging field, these metrics are particularly important due to the high precision required in tasks such as tumor detection and organ delineation. The choice of evaluation metric can significantly impact the perceived performance of a model and guide the development of more effective models. In the following sections, we will delve into some of the most popular and widely used evaluation metrics for semantic segmentation.

#### 3.3.1. Precision

Precision [80], often referred to as the positive predictive value, is a critical evaluation metric in semantic segmentation tasks, particularly in the context of medical imaging. Precision is defined as the ratio of true positive predictions (i.e., the number of pixels correctly identified as belonging to a particular class) to the total number of positive predictions made by the model, which includes both true positives and false positives (i.e., the number of pixels incorrectly identified as belonging to that class). Mathematically, it can be expressed as:

(7) $$Precision=\frac{True\;Positives}{True\;Positives+False\;Positives}$$

Precision provides an estimate of how many of the pixels that the model classifies as belonging to a particular class are actually of that class. A model with high precision demonstrates a low rate of false positives, which is especially valuable in scenarios where the consequences of false positives are significant. However, it is important to note that precision does not take into account false negatives (i.e., pixels of a particular class that the model fails to identify), and thus should be considered alongside other metrics such as recall (or sensitivity) for a more comprehensive evaluation of model performance.

#### 3.3.2. Recall

Recall [80], also known as sensitivity or true positive rate, is another vital evaluation metric in the domain of semantic segmentation, particularly in medical imaging applications. Recall is defined as the ratio of true positive predictions (i.e., the number of pixels correctly identified as belonging to a specific class) to the actual number of pixels that truly belong to that class, which includes both true positives and false negatives (i.e., the number of pixels that are incorrectly identified as not belonging to that class). Mathematically, it can be expressed as:

(8) $$Recall=\frac{True\;Positives}{True\;Positives+False\;Negatives}$$

Recall provides an estimate of the model’s ability to correctly identify all pixels of a particular class. When a model exhibits high recall, it means it has a low rate of false negatives, which is particularly advantageous in situations where avoiding false negatives is crucial. However, it is important to note that recall does not take into account false positives (i.e., pixels that the model incorrectly identifies as belonging to a particular class) and thus should be used in conjunction with other metrics such as precision for a more comprehensive evaluation of model performance.

#### 3.3.3. F-Measure

The F-measure [81], also known as the F1 score, is a widely used evaluation metric in semantic segmentation, particularly in the field of medical imaging. The F-measure is the harmonic mean of precision and recall, two critical metrics that respectively measure the model’s ability to avoid false positives and false negatives. By combining precision and recall, the F_measure provides a single metric that balances the trade-off between these two aspects. Mathematically, it can be expressed as:

(9) $$F\_measure=2\times \frac{Precision\times Recall}{Precision+Recall}$$

The F-measure offers a comprehensive measure of the model’s performance. A high F-measure indicates that both the precision (i.e., the model’s ability to correctly identify pixels of a particular class without incorrectly classifying other pixels as belonging to that class) and the recall (i.e., the model’s ability to correctly identify all pixels of a particular class) are high. This makes the F-measure particularly useful when it is equally important to minimize both false positives and false negatives.

#### 3.3.4. Dice Coefficient

The Dice coefficient [82], also known as the Sørensen–Dice index or Dice similarity coefficient (DSC), is a prominent evaluation metric used in semantic segmentation, particularly in the field of medical imaging. The Dice coefficient measures the overlap between two samples and is particularly useful for comparing the pixel-wise agreement between a predicted segmentation and its corresponding ground truth.

Mathematically, the DSC is calculated by doubling the area of overlap between the predicted and actual ground truth segments and then dividing by the sum of the total pixel counts in both the predicted and ground truth segments.

(10) $$DSC=2\times \frac{Area\;of\;Overlap}{Total\;Number\;of\;Pixels\;in\;Both\;Images}$$

The Dice coefficient provides an estimate of the model’s ability to correctly identify the boundaries of a particular class. A high Dice coefficient indicates a high degree of overlap between the predicted and actual segments, suggesting that the model accurately identifies the boundaries of the class.

#### 3.3.5. Intersection over Union

Intersection over Union (IoU) [82], also known as the Jaccard index, is a commonly used evaluation metric in semantic segmentation, particularly in the field of medical imaging. IoU measures the overlap between the predicted segmentation and the ground truth by dividing the area of overlap by the area of union.

Mathematically, IoU is determined by dividing the area of overlap between the predicted and ground truth segments by the area of their union. It can be expressed as:

(11) $$IoU=2\times \frac{Area\;of\;Overlap}{Area\;of\;Union}$$

IoU provides an estimate of the model’s ability to correctly identify both the location and extent of a particular class. A high IoU indicates a high degree of overlap between the predicted and actual segments, suggesting that the model accurately identifies the location and extent of the class.

#### 3.3.6. Area Under the Curve

The Area Under the Curve (AUC) [81], specifically, the Receiver Operating Characteristic (ROC) curve, is a widely used evaluation metric in semantic segmentation, particularly in the field of medical imaging. The ROC curve represents a graph that demonstrates the diagnostic capability of a binary classification system across varying discrimination thresholds. The Area Under the Curve (AUC) offers a quantification of the model’s proficiency in differentiating between positive and negative classes.

Mathematically, the AUC is the integral of the ROC curve, and it quantifies the overall performance of a model across all possible classification thresholds. An AUC of 1 indicates a perfect classifier, while an AUC of 0.5 suggests a random classifier.

AUC provides an aggregate measure of the model’s performance across different levels of sensitivity and specificity. A high AUC indicates that the model has a high true positive rate for a given false positive rate across various threshold settings.

### 3.4. Medical Image Segmentation Architectures

With the advent of deep learning, a variety of architectures have been proposed and developed specifically for medical image segmentation, offering significant improvements in accuracy and efficiency over traditional methods. In the following sections, we delve into some of the most prominent and widely used architectures for medical image segmentation.

#### 3.4.1. Fully Convolutional Networks

Fully Convolutional Networks (FCNs) [83] have emerged as powerful and efficient approaches to semantic segmentation. FCNs are a class of models within the broader family of convolutional networks, which have been instrumental in driving advances in image recognition tasks. The primary innovation of Fully Convolutional Networks (FCNs) lies in their capacity to process inputs of any size and generate outputs with corresponding dimensions while maintaining efficient inference and learning. A distinctive characteristic of Fully Convolutional Networks (FCNs) is their capability to integrate deep, broad semantic details with shallow, detailed appearance information. This is achieved with a novel architecture that includes in-network upsampling layers, which enable pixel-wise prediction and learning in networks with subsampled pooling. This combination of deep and shallow information allows FCNs to make local predictions that respect the global structure of the image.

**Figure 5:**
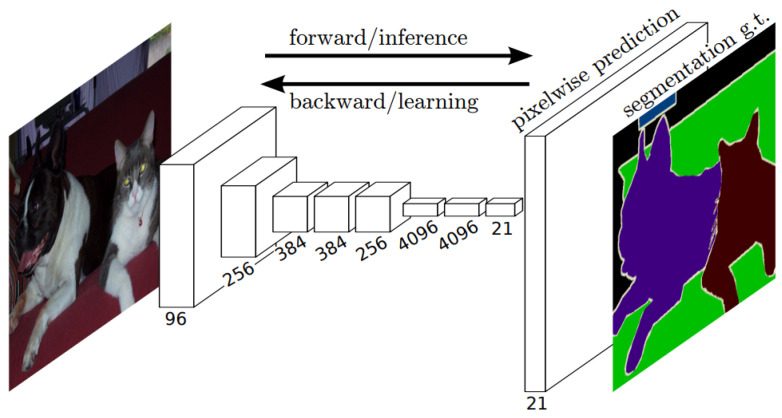
Schematic representation of an FCN [83].

One of the primary advantages of FCNs for semantic segmentation is their ability to provide an end-to-end solution, even when dealing with images of varying sizes. This feature, combined with their other strengths, makes FCNs highly effective tools in the field of semantic segmentation. The limitations of FCNs encompass their substantial computational demands and the challenges they face when adapting to three-dimensional imagery. The architecture of an FCN is depicted in Figure 5.

#### 3.4.2. U-Net

U-Net [84] is another influential architecture that has made significant strides in the field of semantic segmentation, particularly in biomedical image processing. The architecture is designed as an encoder–decoder network, with the encoder extracting features from the input image and the decoder using these features to generate a segmentation map. The U-Net architecture is characterized by its symmetric structure. The encoding path progressively captures the context in the image, while the decoding path helps in precise localization using transposed convolutions. Thus, U-Net combines the advantages of both local features and the global context, which is crucial for accurate semantic segmentation. A distinctive feature of U-Net is the introduction of skip connections between the encoder and decoder. These skip connections allow the network to use information from the earlier layers in the later layers, which helps in recovering the full spatial resolution of the output. This is particularly useful in tasks like medical image segmentation, where every pixel can be important.

**Figure 6:**
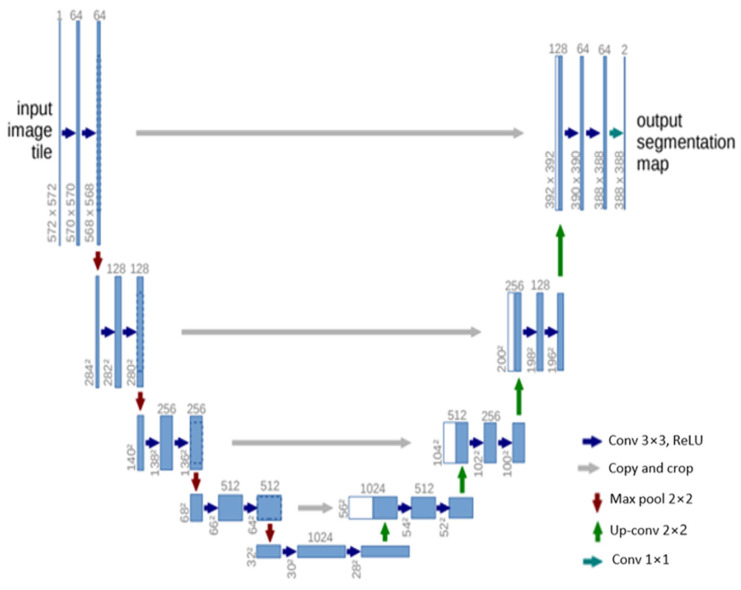
Schematic representation of the U-Net [84].

U-Net offers several advantages for semantic segmentation. It provides an efficient way to deal with the limited amount of annotated images in the medical field, as it can be trained with relatively few images and still deliver high segmentation accuracy. Furthermore, the use of skip connections allows U-Net to capture both high-level and low-level features, leading to more precise segmentation. The structure of the U-Net architecture is demonstrated in Figure 6.

#### 3.4.3. V-Net

**Figure 7:**
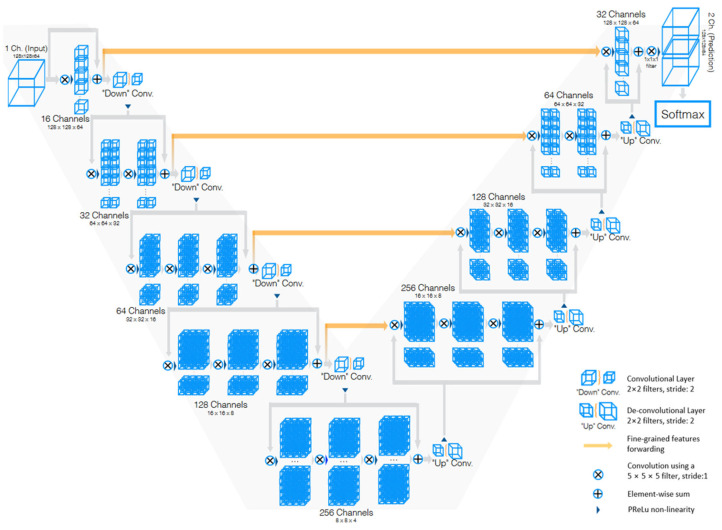
Schematic representation of the V-Net [85].

V-Net [85] is a powerful architecture designed specifically for volumetric medical image segmentation. This refers to a 3D adaptation of the U-Net architecture, specifically crafted to work directly with 3D images. This feature is especially beneficial in medical imaging contexts, where data frequently comprise 3D volumes like MRI or CT scans. The architecture of V-Net is characterized by its symmetric structure. Comparable to the U-Net architecture but with notable distinctions, this network is segmented into various stages, each functioning at different resolutions. Each stage consists of one to three convolutional layers, and a residual function is learned at every stage. Such an architecture enhances the likelihood of convergence compared with networks that do not incorporate residual learning. A distinctive aspect of the V-Net architecture is its use of volumetric kernels in the convolutions executed at each stage. This enables the model to effectively grasp three-dimensional spatial information, essential for precise segmentation in three-dimensional medical imaging. During the compression pathway, the resolution is decreased using convolution with 2 × 2 × 2 voxel-wide kernels, applied with a stride of 2, akin to the pooling layers found in other network architectures. The configuration of the V-Net architecture is depicted in Figure 7.

#### 3.4.4. U-Net++

U-Net++ [86] is an enhanced version of the U-Net architecture, designed to address some of the limitations of the original U-Net. The U-Net++ architecture introduces several innovative features that enhance its performance in semantic segmentation tasks, particularly in the field of medical imaging.

The architecture is characterized by its dense convolutional blocks and deep supervision design. The dense convolutional blocks are designed to reduce the semantic gap between the feature maps of the encoder and decoder, making the learning task easier for the model. These blocks take into account not only the information from the previous nodes in the same level but also the nodes in the level below it. This dense connectivity allows the model to capture both high-level and low-level features, leading to more precise segmentation. The deep supervision design of U-Net++ allows the model to operate in two modes: the accurate mode and the fast mode. In the accurate mode, the outputs from all branches in level 0 are averaged to produce the final result. In the fast mode, not all branches are selected for outputs. This flexibility allows U-Net++ to adapt to different computational and accuracy requirements.

**Figure 8:**
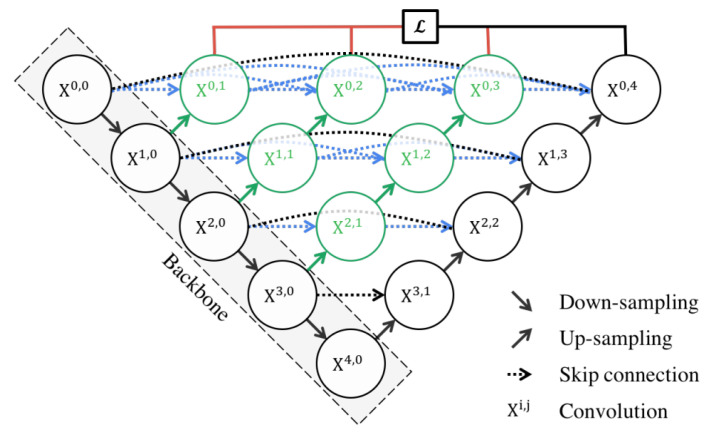
Schematic representation of the U-Net++ [86].

U-Net++ offers several advantages for semantic segmentation. The use of dense connections and deep supervision allows U-Net++ to capture both high-level and low-level features, leading to more precise segmentation. The structure of the U-Net++ architecture is demonstrated in Figure 8.

#### 3.4.5. Attention U-Net

The Attention U-Net [87] presents an innovative approach to image segmentation by incorporating an attention gate (AG) mechanism. This mechanism enables the model to concentrate on specific target structures within an image, thereby enhancing its generalization capability and reducing computational waste on irrelevant activations.

The attention mechanism can be categorized into two types: hard and soft. Hard attention, although effective in highlighting relevant regions, is non-differentiable and necessitates the use of reinforcement learning for training. Conversely, soft attention assigns weights to different regions of the image based on their relevance, allowing for differentiation and training using standard backpropagation. This method ensures that the model focuses more on regions with higher weights during the training process.

The integration of attention into the U-Net architecture addresses the issue of imprecise spatial information during the upsampling phase. U-Net uses skip connections to combine spatial information from both the downsampling and upsampling paths. However, this process often results in the propagation of redundant low-level features. The application of soft attention at these skip connections effectively suppresses activations in irrelevant regions, thereby reducing feature redundancy.

**Figure 9:**
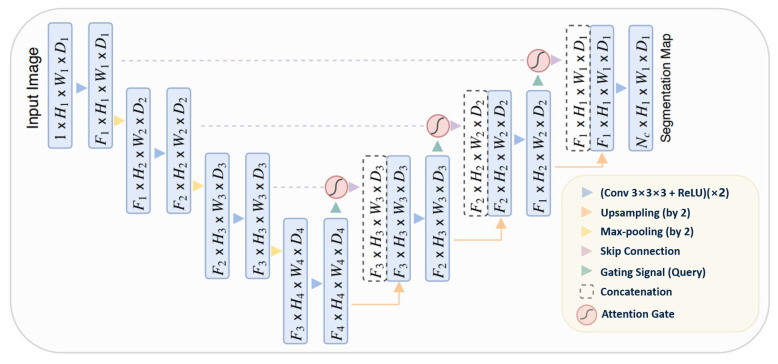
Schematic representation of the Attention U-Net [87].

Attention gates utilize additive soft attention, taking two input vectors: x and g. The vector g, derived from the next lower layer of the network, possesses superior feature representation due to its origin deeper within the network. These vectors undergo an element-wise summation, followed by a ReLU activation layer and a 1 × 1 convolution. The resulting vector is then scaled within the range of [0, 1] through a sigmoid layer, producing the attention coefficients. These coefficients, indicative of feature relevance, are then upsampled to the original dimensions of the x vector and multiplied element-wise to the original x vector, effectively scaling the vector based on relevance. The schematic representation of the Attention U-Net architecture can be seen in Figure 9.

#### 3.4.6. ResUNet++

**Figure 10:**
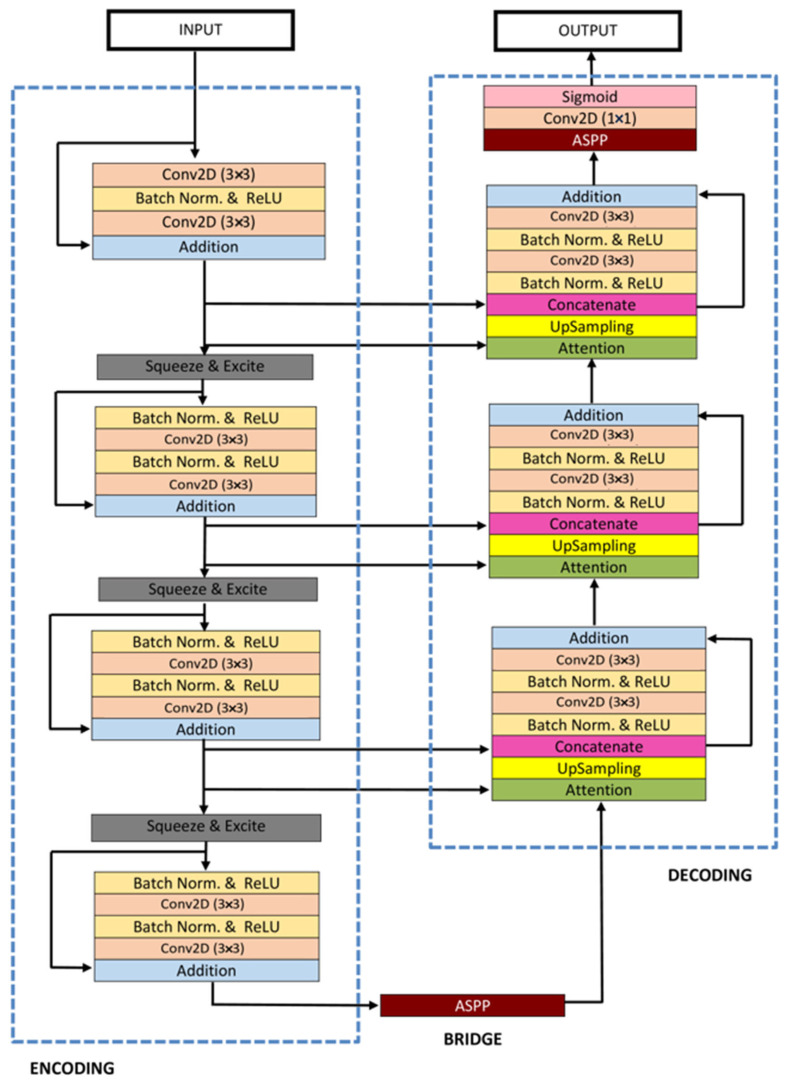
Schematic representation of the Attention ResU-Net ++ [88].

ResUNet++ [88], an enhanced version of the ResUNet architecture, has been developed specifically for medical image segmentation tasks, with a particular focus on pixel-wise polyp segmentation in colonoscopy examinations. The architecture integrates multiple sophisticated elements, such as residual blocks, squeeze and excitation blocks, Atrous Spatial Pyramidal Pooling (ASPP), and attention blocks, enhancing its overall functionality and efficiency. ResUNet++ is based on the Deep Residual U-Net (ResUNet) framework, combining the advantages of deep residual learning with the U-Net structure. Its architecture consists of a stem block, three encoder blocks, an Atrous Spatial Pyramidal Pooling (ASPP) section, and three decoder blocks. In each encoder block of this architecture, there are two consecutive 3 × 3 convolutional blocks followed by identity mapping. A strided convolution layer is then used to reduce the spatial dimensions of the feature maps. Subsequently, the output goes through a squeeze-and-excitation block. The Atrous Spatial Pyramidal Pooling (ASPP) functions as a bridge, offering a wider contextual perspective. The decoding path mirrors the encoding path, utilizing residual units and attention blocks to enhance the effectiveness of feature maps. At the end of the decoder block, its output is routed through the ASPP. Following this, a 1 × 1 convolution using a sigmoid activation function is applied to generate the final segmentation map. The structure of the ResUNet++ model is depicted in Figure 10.

#### 3.4.7. R2U-Net

**Figure 11:**
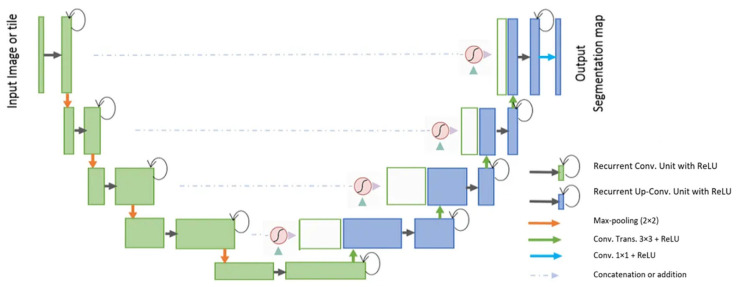
Schematic representation of the Attention R2U-Net [89].

The R2U-Net [89] model incorporates the strengths of deep residual models, Recurrent Convolutional Neural Networks (RCNN), and U-Net to deliver superior performance in segmentation tasks. The architecture of R2U-Net consists of convolutional encoding and decoding units, similar to U-Net. In the R2U-Net architecture, instead of typical forward convolutional layers, Recurrent Convolutional Layers (RCLs) and RCLs integrated with residual units are utilized within both the encoding and decoding units. The incorporation of residual units with RCLs aids in creating a more effective, deeper model. Additionally, this model features an efficient feature accumulation mechanism within the RCL units, ensuring enhanced and more robust feature representation. This is particularly beneficial for extracting very low-level features crucial for segmentation tasks. The R2U-Net distinguishes itself in three key ways: Firstly, it uses RCLs and RCLs with residual units rather than standard forward convolutional layers in both its encoding and decoding stages. Secondly, it incorporates an effective feature accumulation technique within the RCL units, enhancing feature representation. Lastly, it omits the cropping and copying unit found in the basic U-Net model, relying solely on concatenation operations, which leads to a more advanced architecture with improved performance. It also has several advantages over the U-Net model. It is efficient in terms of the number of network parameters. Although R2U-Net maintains the same quantity of network parameters as U-Net and ResU-Net, it demonstrates superior performance in segmentation tasks. The inclusion of recurrent and residual operations in R2U-Net does not augment the total number of network parameters, yet it markedly enhances both training and testing performance. The R2U-Net model’s architecture is visually represented in Figure 11.

#### 3.4.8. nnU-Net

nnU-Net [90] is a robust tool for semantic segmentation that utilizes deep learning to automatically adapt to a given dataset. It simplifies the user experience by analyzing training cases and configuring a corresponding U-Net-based segmentation pipeline, eliminating the need for user expertise in the underlying technology.

The nnU-Net framework encompasses the entire deep learning project pipeline, from preprocessing to model configuration, training, postprocessing, and ensembling. This broad scope makes it a versatile tool for various applications. Its automatic configuration feature addresses critical components like preprocessing and network architecture.

As an open-source tool, nnU-Net delivers state-of-the-art segmentation results right out of the box. Its robustness and self-adapting framework have earned it recognition in the field of medical image segmentation and beyond. Its auto-configuration ability makes it a user-friendly solution for a wide range of segmentation tasks.

## 4. Deep Learning Approaches for Quantifying Ventilation Defects in Hyperpolarized Gas MRI

The application of deep learning techniques to medical imaging has opened new avenues for enhancing diagnostic accuracy and efficiency. This is particularly evident in hyperpolarized gas Magnetic Resonance Imaging, where these advanced computational methods have shown significant promise in quantifying ventilation defects. The following discussion delves into five pioneering research studies that have successfully used deep learning approaches in this context. Each of these studies provides unique insights into the potential of deep learning in improving the analysis and interpretation of hyperpolarized gas MRI data, thereby contributing to our understanding of various pulmonary conditions.

### 4.1. Convolutional Neural Networks and Template-Based Data Augmentation for Functional Lung Image Quantification

In the research paper [48], the authors explore the potential of CNNs in the context of functional lung imaging. The authors present their work on a CNN segmentation framework, specifically tailored for functional lung imaging using hyperpolarized gas. They also address the practical challenges associated with deep learning, such as the need for large training datasets, and propose a novel data augmentation strategy to mitigate this issue. 

#### 4.1.1. Materials and Methods

This study utilized a combination of proton and ventilation images sourced from the authors’ previous and ongoing research. The ventilation images were particularly noteworthy, as they were obtained from both helium-3 and xenon-129 acquisitions. This is significant because the authors’ existing segmentation processing approach [91] did not distinguish between these two types of ventilation gas acquisition protocols, a feature they anticipated would be reflected in their proposed methodology. The acquisition of hyperpolarized MR images was executed in strict adherence to an Institutional Review Board-approved protocol, with each participant providing written informed consent.

MRI data were acquired using a 1.5 Tesla whole-body MRI scanner, equipped with broadband functionality and specialized hyperpolarized gas chest radiofrequency coils. The study utilized two distinct imaging protocols, both of which incorporated hyperpolarized gas and proton imaging acquisitions. The first protocol harnessed 3D balanced steady-state free-precession or spoiled gradient echo pulse sequences, while the second protocol used a contiguous, coronal, 2D gradient echo pulse sequence with an interleaved spiral sampling scheme. In keeping with ethical guidelines, all data were de-identified prior to analysis, thereby ensuring the ethical integrity of the study and the preservation of participant privacy.

In the study, the authors used the U-net architecture to construct distinct models for the segmentation of both structural and functional lung images. In instances where dual acquisition yielded both types of images, the structural images were utilized to generate a mask for the segmentation of the ventilation image. This approach was facilitated with an open-source implementation, developed by the authors’ group and made available through the ANTsRNet Rpackage [92].

Furthermore, the authors implemented a multilabel Dice coefficient loss function, which is a crucial component in evaluating the performance of the segmentation models. They also developed specific batch generators for the generation of augmented image data on the fly. This dynamic approach to data augmentation ensures a diverse and robust training set, enhancing the performance and generalizability of the segmentation models.

#### 4.1.2. Template-Based Data Augmentation

The authors of the study innovatively address a common challenge in deep learning algorithms—the requirement for extensive training datasets. They introduce a novel data augmentation method based on templates, utilizing image data from the population to create an optimal representative template that considers both shape and intensity characteristics. This approach facilitates the transmission of each piece of individual training data across the space of all other training data, effectively enlarging the overall size of the dataset. During model training, a new augmented data instance is created by mapping a randomly selected source subject to the space of a target subject. This approach enables the expansion of a training dataset of size N to a dataset of size N^2^. The authors also discuss a variant of this method, where a template is built from M datasets (where M > N), resulting in an augmented dataset size of M × N.

**Figure 12:**
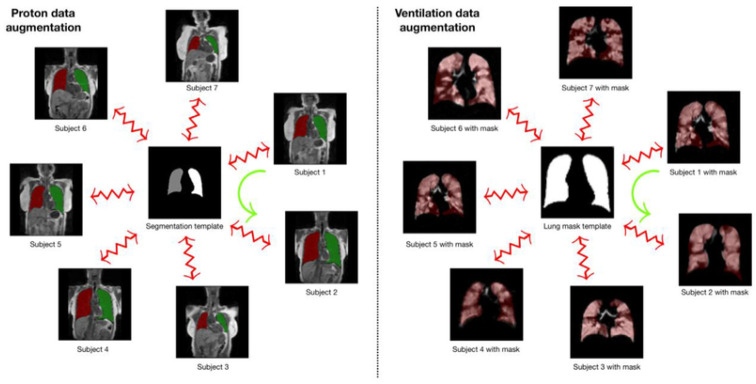
The template-based data augmentation process used in the creation of proton (**left**) and ventilation (**right**) U-net models. A template is established and used to generate transformations for each subject. These transformations, which are deformable and invertible, for the k_th_ subject, S_k_ to the template, T, are denoted by φ_k_: S_k_↔T and are utilized during model training. The data augmentation process involves randomly selecting a reference and a target subject during batch processing. As an example, the transformation from Subject 1 to the space of Subject 2, represented by the green curved arrow, is defined as φ_2_^−1^(φ_1_) [48].

The authors applied this template-based data augmentation separately for proton and ventilation data, creating distinct templates for each. They used 60 proton MR images, allowing for 3600 potential deformable shapes, which could be further augmented using conventional strategies. Similarly, a ventilation template was created from the training ventilation images. This innovative approach to data augmentation signifies a substantial contribution to the field of medical image segmentation. Figure 12 illustrates the process of template-based data augmentation for generating both the proton (on the left) and ventilation (on the right) U-net models.

#### 4.1.3. Deep Learning-Based Segmentation Architecture

**Figure 13:**
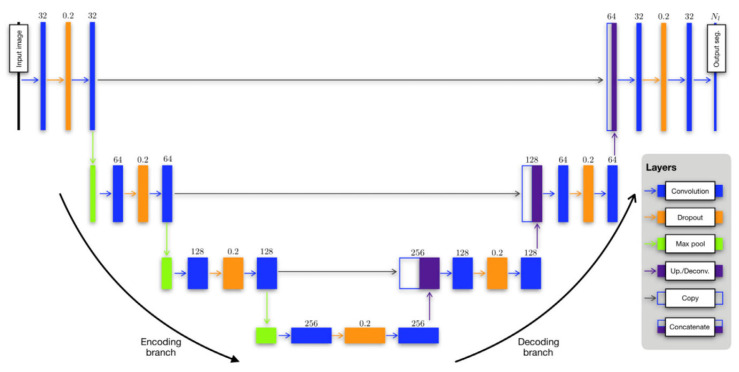
The adapted U-net framework for both structural and functional lung segmentation [48].

In their study, the authors used a dataset comprising 205 proton MR images, each accompanied by left/right lung segmentations, and 73 ventilation MR images with corresponding masks. Separate U-net models were trained for these images. The dataset underwent denoising and bias correction, a process that required less than a minute per image. The authors utilized an R script to read the images and segmentations, establish the model, configure model parameters, and initialize the batch generator. To handle the proton data, a 3D U-net model was constructed, capitalizing on the distinctive 3D shape of the lungs. However, this approach faced limitations due to the GPU’s memory capacity. The authors acknowledged that this limitation could potentially be addressed with additional computational resources. In the batch generator, transforms derived from the template-building process described earlier were utilized for the proton data. Random assignments of reference and source subjects were made, and during each iteration, these random pairings were used to generate augmented data. The U-net ventilation model was developed using 73 ventilation MRIs, and the reduced dataset size was a consequence of data pruning to achieve class balance. Although the functional images were processed as 3D volumes and a 3D ventilation template was created for template-based data augmentation, the resulting U-net model was 2D. This choice was guided by factors such as shorter training and prediction times for 2D models compared with 3D models. Additionally, it was determined that 2D models were sufficient for functional lung imaging, as existing state-of-the-art methods lacked complex shape priors. The modified U-net is depicted in Figure 13.

The authors highlighted that segmentation of any ventilation image would be conducted on a slice-by-slice basis, with each slice being segmented using the 2D model. Regarding data augmentation, complete 3D transforms were provided to the batch generator. During each iteration, a set of 3D augmented images was dynamically generated, and a subset of slices was randomly chosen for each image until the batch set was fully formed. The training process for both the 3D U-net structural model and the 2D U-net functional model was relatively time-intensive, requiring approximately 10 h in total. However, once the models were constructed, the prediction time was remarkably efficient, taking less than a second per image. Both the training and prediction processes were performed using an NVIDIA Titan Xp GPU. A summary of their configured U-Net architecture is provided in Table 1. 

**Figure 14:**
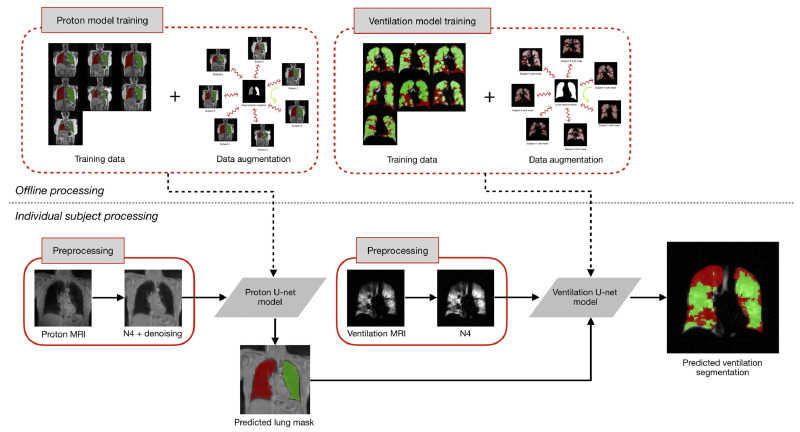
The training of U-net models for both proton and ventilation imaging incorporates template-based data augmentation. This offline training requires significant computational resources but is executed only once. The subsequent preprocessing for each subject involves MR noise reduction and bias correction. The proton mask, derived from the proton U-net model, is incorporated as an additional channel for ventilation image processing [48].

An illustration of the proposed workflow is provided in Figure 14.

#### 4.1.4. Results

In the referenced study, the researchers implemented the U-net structural model, developed using template-based data augmentation, on a test dataset comprising the same 62 proton MRIs utilized in a prior investigation. The performance of this U-net model was subsequently juxtaposed with a refined version of the Joint Label Fusion (JLF) [93] technique, a method currently in use in their ongoing studies.

The refinement of the JLF method involved a strategic deviation from the use of the complete atlas set, which would necessitate a substantial number of pairwise image registrations. Instead, the center of the image under segmentation was aligned with each atlas image, followed by the computation of a neighborhood cross-correlation similarity metric. The ten most similar atlas images were then selected for the JLF scheme.

The comparative performance of the two methodologies was assessed using the Dice overlap metric. The U-net model demonstrated a Dice overlap of 0.93 ± 0.03 for the left lung, 0.94 ± 0.02 for the right lung, and 0.94 ± 0.02 for the whole lung. The refined JLF method, while achieving marginally higher Dice overlap scores (0.95 ± 0.02 for the left lung, 0.96 ± 0.01 for the right lung, and 0.96 ± 0.01 for the whole lung), required significantly more processing time. The U-net model required less than a second per subject for processing, in contrast with the JLF method, which demanded approximately 25 min per subject. This time difference persisted even when the JLF method utilized four CPU threads to execute eight parallel pairwise registrations for each evaluation image.

As for the results of the ventilation lung MRI lung segmentation, the researchers implemented their deep learning methodology on the evaluation data from their previous work. The probability images generated as output were combined into a unified segmentation image. This segmentation image was subsequently compared to the manual segmentation results and the outcomes produced by Atropos, a method introduced in their previous work. Notably, they excluded Otsu thresholding and K-means thresholding due to their subpar performance and disregard for spatial information, a feature that both computational methods and human readers consider.

In the absence of a definitive ground truth, the researchers used the STAPLE algorithm to create consensus labeling. To assess performance, they utilized the Dice overlap coefficient to measure the agreement between each segmentation rater and the consensus labeling. Below are the mean values for total lung, normal lung, and ventilation defect from the results:

- Reader 1 recorded values of 0.89 ± 0.07 for total, 0.91 ± 0.06 for normal lung, and 0.6 ± 0.3 for ventilation defect.
- Reader 2 reported values of 0.92 ± 0.05 for total, 0.94 ± 0.04 for normal lung, and 0.57 ± 0.3 for ventilation defect.
- Reader 3 observed values of 0.94 ± 0.03 for total, 0.96 ± 0.03 for normal lung, and 0.63 ± 0.3 for ventilation defect.
- Atropos yielded results of 0.92 ± 0.03 for total, 0.94 ± 0.03 for normal lung, and 0.71 ± 0.3 for ventilation defect.
- U-net generated values of 0.94 ± 0.03 for total, 0.96 ± 0.03 for normal lung, and 0.70 ± 0.3 for ventilation defect.

**Figure 15:**
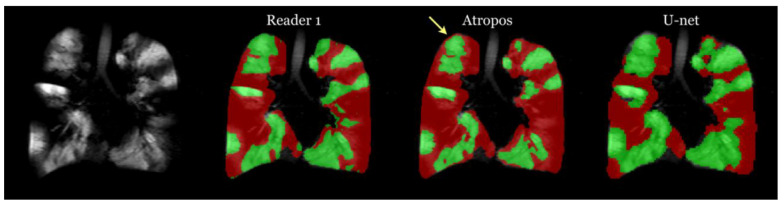
Comparison of ventilation segmentation between manual human interpretation and the two computational methodologies. Observe the impact of partial volume effects at the lung apex, marked by the yellow arrow, which the Atropos method erroneously classifies as a ventilation defect. In contrast, the U-net method and the human evaluator accurately identify this area [48].

The processing time varied significantly across methods: Atropos required less than a minute per subject, human readers took between 30 and 45 min, and the U-net model processed each subject in less than a second. This highlights the remarkable processing efficiency of the U-net model. Figure 15 presents a comparison of ventilation segmentation between manual human interpretation and the two computational methodologies.

### 4.2. 3D Deep Convolutional Neural Network-Based Ventilated Lung Segmentation Using Multi-Nuclear Hyperpolarized Gas MRI

In the study [94], Astley et al. present a novel deep learning approach to enhance the segmentation of ventilated lung regions in MRI scans. Utilizing hyperpolarized gases such as ^3^He and ^129^Xe, the authors use a 3D deep convolutional neural network to accurately delineate ventilated areas. This method stands out for its ability to handle the high-dimensional data inherent in hyperpolarized gas MRI, providing a significant advancement over traditional segmentation techniques. 

The paper details a meticulous training process using the NiftyNet [95] framework and TensorFlow [96], optimized using Adam [77] with carefully tuned learning rates and batch sizes to maximize the efficacy of the CNN. The authors’ approach demonstrates a marked improvement in segmentation accuracy when compared with conventional methods, as evidenced by their comprehensive testing across five different experimental setups. Notably, the combination of ^3^He and ^129^Xe scans in the training set emerged as the most effective strategy, highlighting the potential of multi-nuclear imaging data in enhancing deep learning models. 

#### 4.2.1. Materials and Methods

The study involved 3D volumetric MRI scans using hyperpolarized ^3^He and ^129^Xe gases, conducted on a 1.5T MRI system. For signal transmission and reception, flexible quadrature radiofrequency coils tuned to the respective Larmor frequencies of ^3^He and ^129^Xe were used. The scans achieved an in-plane resolution of 4 × 4 mm^2^. The ^129^Xe MRI scans varied from 16 to 34 slices, averaging 23 slices with a slice thickness of 10 mm. In contrast, the ^3^He MRI scans had a range of 34 to 56 slices, with an average of 45 slices and a thinner slice profile at 5 mm.

The dataset compiled for the study was retrospectively collected from various clinical observational studies and patients who underwent clinical scans. It encompassed a total of 743 volumetric hyperpolarized gas MRI scans, which included 248 scans (11,370 slices) using ^3^He and 495 scans (11,520 slices) using ^129^Xe, from 326 subjects. The distribution of slices was roughly equal between the two gases. 

Every MRI scan was paired with a meticulously hand-crafted ground truth segmentation of the ventilated lung regions. These segmentations underwent a rigorous process, involving multiple expert observers’ meticulous editing and subsequent review by an independent imaging scientist. This comprehensive approach was taken to guarantee the accuracy and consistency of the segmentations. In the event of any identified errors, such as the unintentional inclusion of the trachea or background noise within the segmentations, manual corrections were diligently applied. This meticulous attention to detail was essential to preserve the dataset’s integrity and accuracy.

#### 4.2.2. Deep Learning-Based Segmentation Architecture

The study utilized a V-Net [85] fully convolutional neural network, which processes 3D scans using volumetric convolutions. The network used PReLu [69] activation functions and binary cross-entropy loss [97] for optimization. The Adam optimizer was harnessed with a defined spatial window size and batch size. The learning rate underwent adjustments during both the initial training and fine-tuning stages of the model. Training and inference were executed on an NVIDIA Tesla V100 GPU using the NiftyNet framework on TensorFlow. The researchers systematically conducted a series of experiments to assess the influence of various network architectures, loss functions, and pre-processing methods. These experiments were carried out on a subset of 431 hyperpolarized gas MRI scans, encompassing both healthy subjects and patients with pulmonary conditions. Out of these scans, 29 were dedicated to parameterization testing, while 40 were utilized for internal validation. Notably, the V-Net architecture, coupled with the cross-entropy loss function, demonstrated outstanding performance, as evidenced by its superior Dice similarity coefficient, average Hausdorff distance, and Hausdorff 95th percentile distance. Pre-processing techniques like normalization and denoising were tested but did not yield significant improvements, leading to their exclusion from the larger dataset.

Five distinct experimental methods were used to train the network on different combinations and iterations of ^3^He and ^129^Xe scans. These methods were designed to determine the most effective training process across various metrics, with each method being rigorously compared using the same testing set.

#### 4.2.3. Results

The study used five different deep learning training strategies to optimize the network’s performance:

- Training solely on 232 scans with ^3^He gas for 25,000 iterations.
- Training solely on 437 scans with ^129^Xe gas for 25,000 iterations.
- Initial training on 232 scans with ^3^He gas for 20,000 iterations, followed by further training on 437 scans with ^129^Xe gas for 5000 iterations using the weights from the first training phase.
- Initial training on 437 scans with ^129^Xe gas for 20,000 iterations, followed by further training on 232 scans with ^3^He gas for 5000 iterations using the weights from the first training phase.
- Combined training on 669 scans with both ^3^He and ^129^Xe gases for 25,000 iterations.

In the assessment of segmentation performance across various deep learning experimental methods, the approach that integrated training on both ^3^He and ^129^Xe isotopes demonstrated superior results. This method achieved a DSC of 0.958, an average Hausdorff distance (Avg HD) of 2.22 mm, a 95th percentile Hausdorff distance (HD95) of 8.53 mm, and an XOR metric of 0.087. These values were the highest recorded for the respective metrics among the tested methods. In contrast, traditional segmentation methods such as K-means and SFCM lagged, with K-means recording significantly higher values of Avg HD (37.28 mm) and HD95 (98.79 mm), indicating less precise segmentation. The other DL methods varied in performance, with individual methods trained exclusively on either ^3^He or ^129^Xe showing competitive results but not exceeding the combined approach. Fine-tuning across isotopes also influenced performance, with slight variations in metric outcomes.

**Figure 16:**
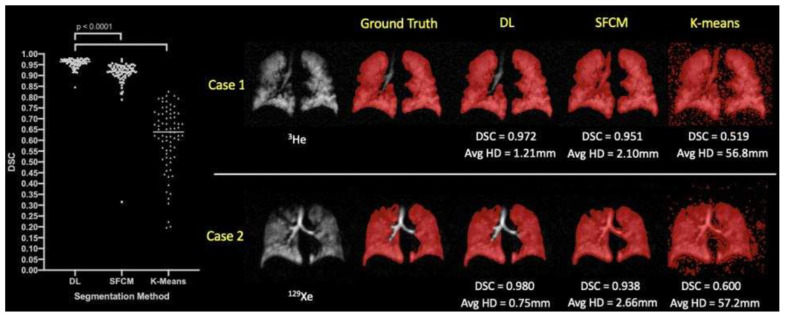
Comparative analysis of segmentation accuracy in hyperpolarized gas MRI. The deep learning (DL) method using combined ^129^Xe and ^3^He data is contrasted with standard segmentation techniques (SFCM and K-means). Statistical significance is denoted with *p*-values from paired *t*-tests. For two individual cases—Case 1 representing a COPD patient and Case 2 a clinical referral patient—the Dice similarity coefficient (DSC) and average Hausdorff distance (Avg HD) metrics are presented for each segmentation method [94].

When evaluating the segmentation performance of deep learning methods stratified by disease, the data indicated that training on ^129^Xe exclusively provided the best result for healthy subjects with a mean DSC of 0.952. For lung cancer patients, training on ^3^He and fine-tuning on ^129^Xe was most effective, achieving a mean DSC of 0.955. Patients with COPD showed the highest mean DSC of 0.968 when trained on ^129^Xe, fine-tuned on ^3^He. In cases of CF, combined training on ^3^He and ^129^Xe yielded a mean DSC of 0.956. The segmentation for premature children was best when trained on ^3^He, fine-tuned on ^129^Xe with a mean DSC of 0.929. For Interstitial Lung Disease (ILD), training on ^129^Xe alone was most effective with a mean DSC of 0.959. Lastly, clinical referrals saw the highest mean DSC of 0.961 with combined ^3^He and ^129^Xe training. These results showcase the effectiveness of tailored DL training approaches for different diseases in improving segmentation accuracy. A comparison of performance on testing scans is provided in Figure 16.

The study concludes that deep learning techniques excel in segmenting lung regions from hyperpolarized gas MRI scans, with a large and diverse dataset enhancing the model’s accuracy and generalizability. The combined use of ^3^He and ^129^Xe scans in training improved the model’s performance, achieving a high Dice similarity coefficient. While the study notes limitations such as potential observer variability and an imbalance in the dataset, future research aims to refine the model further. 

### 4.3. Large-Scale Investigation of Deep Learning Approaches for Ventilated Lung Segmentation Using Multi-Nuclear Hyperpolarized Gas MRI

In the study [47], the authors conducted a thorough series of experiments aimed at identifying the most efficient 3D CNN architecture, loss function, and pre-processing techniques for hyperpolarized gas MRI segmentation. Their research encompassed the evaluation of five distinct deep learning methods, all implemented with the optimal configuration. The ultimate goal was to attain precise, resilient, and rapid segmentation of ventilated lungs in hyperpolarized gas MRI scans.

The performance of these methods was assessed using a variety of evaluation metrics and a diverse testing set, which included both ^3^He and ^129^Xe noble gas scans along with corresponding expert segmentations. The authors also explored the impact of the type of noble gas on the performance of deep learning. 

In addition, they compared the top-performing deep learning method with traditional approaches. Lastly, they assessed the correlation and agreement of ventilated lung volume for the best-performing deep learning method in comparison to volumes derived from expert segmentations.

#### 4.3.1. Materials and Methods

In the study, all participants underwent 3D volumetric hyperpolarized gas MRI scans using either ^3^He or ^129^Xe. These scans were carried out on a 1.5 Tesla HDx scanner, utilizing 3D steady-state free precession sequences. The MRI signals were transmitted and received using flexible quadrature radiofrequency coils tuned to the Larmor frequencies of the respective gases. The in-plane resolution for both types of scans was set at 4 × 4 mm^2^. The ^129^Xe scans consisted of between 16 and 34 slices, averaging 23 slices, with a slice thickness of 10 mm. On the other hand, the helium-3 scans ranged from 34 to 56 slices, averaging 45 slices, with a slice thickness of 5 mm.

The study utilized a dataset compiled retrospectively from various research and clinical studies involving patients who underwent hyperpolarized gas MRI scans. The dataset, approved for use by the Institutional Review Boards at the University of Sheffield and the National Research Ethics Committee, was fully anonymized and complied with all relevant guidelines and regulations. It comprised 759 volumetric hyperpolarized gas MRI scans, split almost evenly between ^3^He and ^129^Xe scans, from 341 subjects. These subjects included both healthy individuals and patients with a range of pulmonary conditions. Each scan in the dataset was paired with an expert-edited segmentation representing the ventilated region of the lungs. These segmentations, collected from numerous retrospective studies, were generated using various semi-automated methods and edited by multiple expert observers. An experienced imaging scientist conducted quality control, manually correcting potential errors to ensure segmentation accuracy and removing any voxels outside of the lung parenchymal region defined by a structural ^1^H MRI scan.

#### 4.3.2. Deep Learning-Based Segmentation Architecture

**Figure 17:**
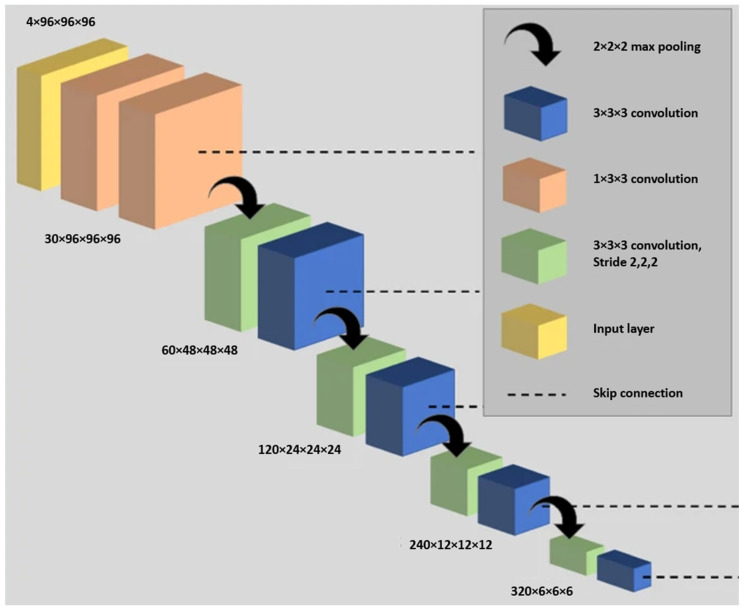
The adapted 3D nn-UNet network used in the referenced study. The deconvolution side, which parallels the convolutional path and includes an extra 1 × 1 × 1 SoftMax layer, is not depicted in the figure [47].

The research carried out extensive parameterization experiments with the aim of pinpointing the most efficient 3D CNN architecture, loss function, and pre-processing techniques for hyperpolarized gas MRI segmentation. In this study, the researchers used nn-UNet [90], a fully convolutional neural network, to process 3D scans with volumetric convolutions. The network was trained end-to-end using hyperpolarized gas MRI volumetric scans. They utilized a 3D version of the U-Net, which was modified to alleviate memory constraints, thereby allowing for 30 feature channels. The size of the convolution operations varied from 3 × 3 × 3 to 1 × 1 × 1, contingent on the network layer. A visual representation of the modified 3D nn-UNet network is provided in Figure 17. Each hyperpolarized gas MRI scan underwent a pre-processing phase using spatially adaptive denoising. This method was specifically designed to account for both Rician noise and spatially varying noise patterns. The denoising process was implemented using the DenoiseImage function in ANTs 2.1.0 across three dimensions, with standard parameters applied. For data augmentation, the researchers used constrained random rotation and scaling. The rotation was limited to a range of −10° to 10°, and scaling was within −10% to 10%. These random rotations or scalings were applied within these specified limits. Each rotation axis and scaling factor were assigned a unique random value. Table 2 summarizes the network configurations.

The dataset was divided into two subsets: a training set comprising 75% of the data and a testing set containing the remaining 25%. The training set comprised 237 ^3^He scans and 436 ^129^Xe scans, while the testing set included 86 scans, each from a different subject. The researchers ensured that there was no overlap between the subjects in the training and testing sets.

The researchers conducted five distinct deep learning experimental methods to train the network, as listed below:

1. Training on 237 ^3^He scans for 30,000 iterations.
2. Training on 436 ^129^Xe scans for 30,000 iterations.
3. Training on 237 ^3^He scans for 20,000 iterations, then fine-tuning on 436 ^129^Xe scans for 10,000 iterations.
4. Training on 436 ^129^Xe scans for 20,000 iterations, then fine-tuning on 237 ^3^He scans for 10,000 iterations.
5. Training on both 436 ^129^Xe and 237 ^3^He scans for 30,000 iterations.

Each of the networks underwent training using the NiftyNet framework designed for medical imaging deep learning. These training sessions, as well as the subsequent inference processes, were carried out on an NVIDIA Tesla V100 GPU equipped with 16 GB of RAM.

#### 4.3.3. Deep Learning vs. Conventional Methods

During the comparison to conventional methods, the researchers evaluated their deep learning approach against two traditional machine learning methods, namely, Spatial Fuzzy C-Means (SFCM) [98] and K-means clustering [36]. The K-means algorithm is commonly used in hyperpolarized gas MRI segmentation to group data points into clusters centered around their respective centroids. In contrast, the SFCM method uses a bilateral filter to reduce noise while preserving edges, considering spatially close pixels with high correlation to belong to the same cluster. It incorporates voxel membership within a defined window and adjusts the central pixel based on weighting variables. The performance of each of the five deep learning experimental approaches, as well as the two traditional methods, was assessed using a variety of metrics on the test dataset, which is listed in Table 3.

#### 4.3.4. Results

A variety of statistical tests was used to analyze the data. The normality of the data was first assessed using Shapiro–Wilk tests. In cases where normality was not met, non-parametric tests were utilized. To assess the statistical significance of variations among the different experimental deep learning methods, either one-way repeated-measure ANOVA or Friedman tests were performed, with post hoc multiple comparisons adjusted using the Bonferroni correction. To assess the impact of the noble gas used (either ^3^He or ^129^Xe) on the segmentations in the testing set, independent *t*-tests or Mann–Whitney U tests were used. The top-performing DL method was compared to traditional segmentation approaches using either one-way repeated-measure ANOVA or Friedman tests, with post hoc multiple comparisons adjusted using the Bonferroni correction. To compare the volumes of DL-generated and expert segmentations, Pearson or Spearman correlations were calculated and Bland–Altman analysis was conducted. All statistical analyses were carried out using Prism 8.4 (GraphPad, San Diego, CA, USA) and SPSS Statistics 26.0 (IBM Corporation, Armonk, NY, USA). Five deep learning methods were applied to generate segmentations for 86 test scans. These scans included both healthy subjects and patients with various pulmonary pathologies, and the segmentations were created using either ^3^He or ^129^Xe. The quality of these segmentations was evaluated by comparing them with the original scans and expert segmentations. The DL methods were generally successful in excluding voxels outside the lung parenchyma and accurately identifying ventilation defects. One interesting case involved a healthy subject with a zipper artifact caused by electronic noise in the hardware. Some models were able to accurately exclude this artifact, while others could not distinguish between the artifact and ventilated lung voxels.

**Figure 18:**
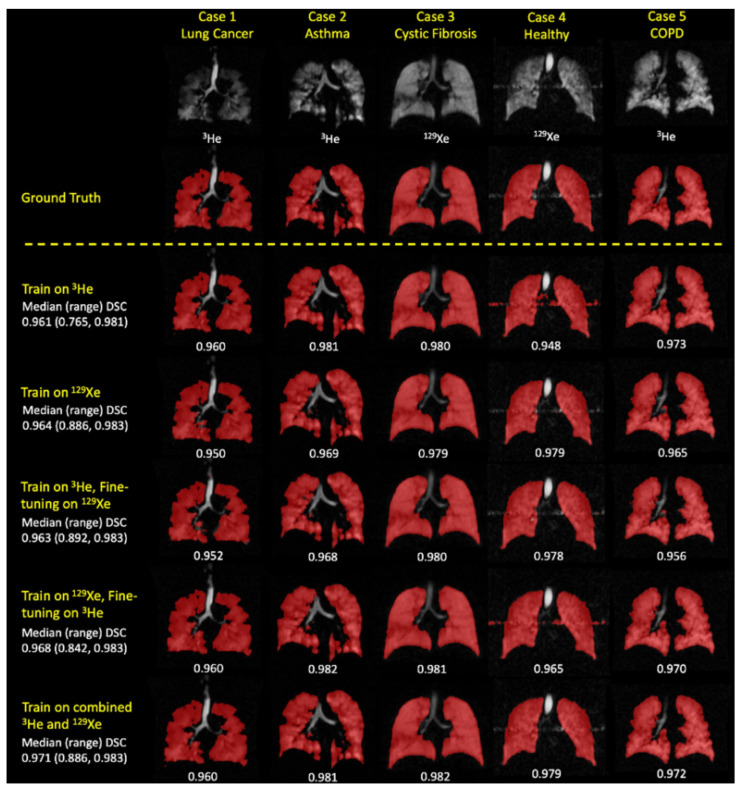
Sample coronal slices for a healthy individual and four cases with distinct pathologies, each corresponding to a different deep learning experimental approach. The figure includes individual DSC values, as well as median (range) DSC values [47].

Figure 18 illustrates the quality of segmentation for a healthy individual and patients with four distinct pathologies, utilizing five different deep learning experimental methods with either ^3^He or ^129^Xe.

The combined ^3^He and ^129^Xe method was identified as the best-performing method, generating the most accurate segmentations across all four metrics. This method also showed statistically significant improvements over all other DL methods in the DSC, exclusive OR (XOR), and 95th percentile Hausdorff distance (HD95) metrics.

The study evaluated the combined ^3^He and ^129^Xe DL model on 31 2D spoiled gradient-echo ^3^He hyperpolarized gas MRI ventilation scans with varying MRI sequences and acquisition parameters. The findings demonstrated that the model exhibited strong generalization, providing consistent and high-quality segmentations regardless of the specific MRI sequence used.

Additionally, the study examined the ventilated volume using the combined ^3^He and ^129^Xe method. The DL segmentation volume showed a strong correlation (r = 0.99) with the expert segmentation volume and displayed minimal bias, with a deviation of only −0.8%. The study also compared the performance of the DL methods with two conventional segmentation methods, namely, K-means clustering and Spatial Fuzzy C-Means (SFCM). The DL segmentation method exhibited significant improvements over these conventional methods, accurately excluding low-level noise and artifacts, as well as non-lung regions such as the trachea and bronchi.

**Figure 19:**
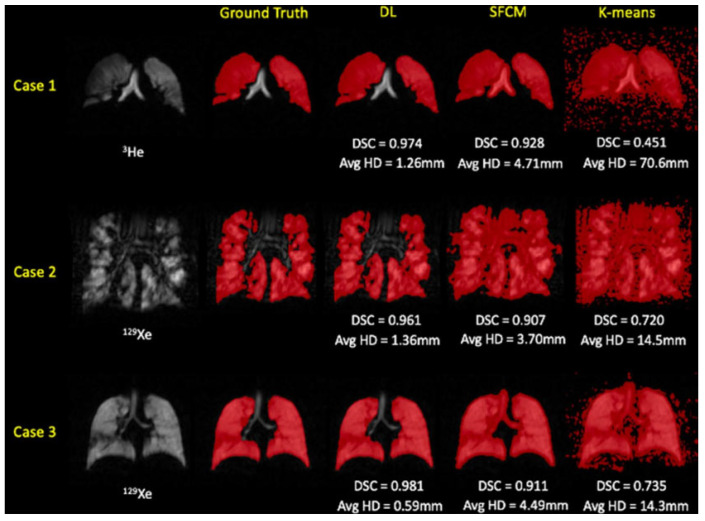
Evaluating the performance of the combined ^129^Xe and ^3^He deep learning method in comparison to traditional segmentation methods (SFCM and K-means) on test scans [47].

Figure 19 presents a qualitative comparison of the combined ^3^He and ^129^Xe deep learning methods with two traditional segmentation methods (K-means and SFCM) across three cases.

The study showcased the effectiveness of deep learning segmentation methods in processing a large dataset of 759 scans from patients with diverse lung pathologies. The combined ^3^He and ^129^Xe DL method outperformed all other methods across various metrics, demonstrating its unbiased performance irrespective of the noble gas used. This is particularly relevant given the global shift toward the use of ^129^Xe due to the scarcity of ^3^He.

The CNN outperformed traditional methods across all evaluation metrics, effectively addressing challenges like background noise and artifacts while accurately excluding ventilation defects and airways. This hints at the possibility of reducing post-segmentation manual editing time. The study emphasized the importance of additional validation for DL-based methods, not only considering ventilated lung volume but also VDP for clinical applicability. Furthermore, CNN-generated segmentations demonstrated a strong correlation and minimal bias compared with expert volumes in estimating ventilated lung volumes.

### 4.4. Quantification of Lung Ventilation Defects on Hyperpolarized MRI: The Multi-Ethnic Study of Atherosclerosis (MESA) COPD Study

In the research paper titled “Quantification of lung ventilation defects on hyperpolarized MRI: The Multi-Ethnic Study of Atherosclerosis (MESA) COPD study” [49] by Xuzhe Zhang et al., the authors developed a deep learning framework for the segmentation of lung MRI scans. The approach uses a cascaded U-Net model to segment both proton (^1^H) and hyperpolarized ^3^He MRI scans, distinguishing among non-ventilated, hypo-ventilated, and standard ventilated areas. A key aspect of their methodology is the use of data augmentation techniques, which increased the diversity of the training data and improved the model’s robustness. The authors tested this model on participants with and without COPD and found its performance to be similar across both groups. The research also highlights the robustness of the model in quantifying ventilation defects, which was improved with the use of conventional data augmentation and the cascaded U-Net. The authors innovatively used a pre-trained generative adversarial network (GAN) for data augmentation, creating a realistic synthetic dataset. This approach is particularly beneficial in scenarios where the available training data are limited, as it allows for the generation of additional, synthetic training samples that can enhance the learning capabilities of the model. The use of GANs for data augmentation is a cutting-edge technique that has shown promise in various applications, including medical imaging. The synthetic data generated using the GAN closely mimics the distribution of the real data, thereby providing the model with a broader range of data to learn from and potentially improving its performance. The authors acknowledged the limitations of their study, including the manual selection of regions of interest (ROIs) for threshold determination and the potential propagation of errors from original to augmented data. 

#### 4.4.1. Materials and Methods

The study under review utilized data from the MESA COPD Study [99], focusing on smokers with a significant smoking history. A subset of these participants underwent simultaneous ^1^H and ^3^He MRI scans. The researchers selected 9–10 registered ^1^H and ^3^He MRI coronal slices per participant, which were then divided into training and test datasets in a 3:1 ratio. The majority of the slices were used for training, with a portion set aside for validation. The remaining slices were used for testing. The study was conducted in compliance with HIPAA rules and received the necessary approvals.

The ^3^He MRI was conducted using a 3 T Achieva Philips MR scanner, complemented with a flexible wrap-around ^3^He radio frequency coil. The ^3^He gas was polarized to an average of 29%, facilitated with a ^3^He polarizer from GE Healthcare. 

The investigation yielded 544 slices of both ^1^H and ^3^He pulmonary MRI scans, obtained from 56 participants. Each slice underwent annotation and semi-automatic segmentation. The creation of a reliable ground-truth method was made possible with the collaborative efforts of the Image Analysis Core Lab, encompassing experienced image analysts, radiologists, physiologists, and physicians.

The researchers used a detailed method for annotating full lung masks, using coronal^1^H MRI images from each participant to precisely define lung boundaries. The study focused on a specific region of interest (ROI) in each coronal slice of the lungs, deliberately omitting areas influenced by partial volume effects. For enhanced accuracy, they computed the average and standard deviation of the lung ROI’s signal intensity in each coronal slice. Lung segmentation in ^1^H MRI images was then conducted using a region-growing technique, finely adjusted to thresholds based on the lung ROI’s mean signal intensity and its variation. This ensured a reliable and precise segmentation process. Additionally, manual refinements were made where needed, mainly for subtle anatomical adjustments including the exclusion of low-intensity areas such as cortical bones, central airways, and the background. 

The study used a thorough process to annotate ventilation in lung scans. Lung masks derived from ^1^H MRI images were utilized on corresponding ^3^He slices to establish lung boundaries. The signal intensity (SI) from the heart and central airways served as benchmarks for identifying non-ventilated and ventilated regions, respectively. On-ventilated regions were segmented using a region growing method. Regions with ventilation were subsequently classified into two distinct types: those with normal ventilation and those with hypo-ventilated, using designated threshold values for differentiation. Analysts, blinded to the participants’ clinical information, performed the image analysis. Inter-reader and intra-reader agreements were evaluated using ten randomly selected MRI scans. For manual selection of ROI, region expansion, and corrections, the study utilized the SliceOmatic tool, developed by TomoVision in Magog, Canada.

#### 4.4.2. Deep Learning-Based Segmentation Architecture

The deep learning framework incorporated separate ^1^H and ^3^He segmentation models, each trained on their respective MRI datasets. The developed framework was engineered for ventilation defect segmentation using supervised training. In this process, annotated full lung masks from the ^1^H MRI were fed into the ^3^He model accompanied by the ^3^He MRI data.

The framework was built as an end-to-end system for testing purposes. Initially, the ^1^H MRI underwent segmentation using the ^1^H segmentation model. The segmented full lung masks obtained from this step were then integrated with the ^3^He MRI data, serving as input for the ^3^He segmentation model. 

**Figure 20:**
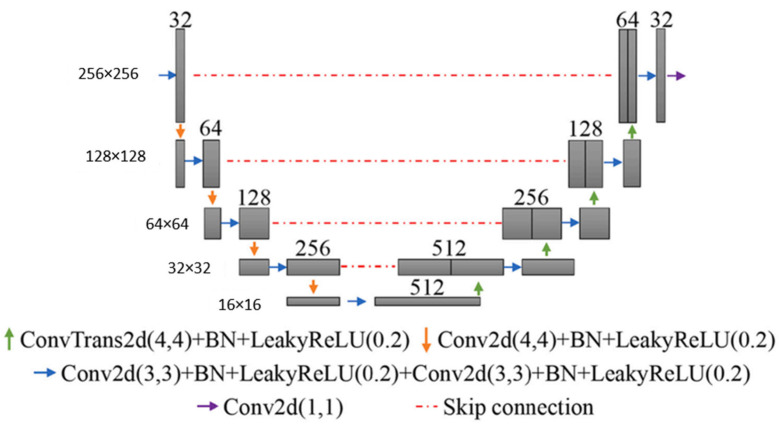
The modified U-Net architecture used in the referenced study [49].

The U-Net architecture was utilized to build both the ^1^H and ^3^He segmentation models. The ^1^H segmentation model utilized a modified U-Net, substituting its max pooling layer with a 2D convolution layer for generating full lung masks. In contrast, the ^3^He segmentation model used a cascaded U-Net. This cascaded architecture used full lung masks and ^3^He MRI scans as dual-channel input, initially segmenting into non-ventilated and ventilated areas. The segmented ventilated areas were then processed further to distinguish between hypo-ventilated and normal-ventilated regions. Both ^1^H and ^3^He MRI image intensities were normalized within a range of [−1, 1]. Figure 20 demonstrates the U-Net structure, highlighting the replacement of the max pooling layer with a 4 × 4 kernel 2D convolution layer.

**Figure 21:**
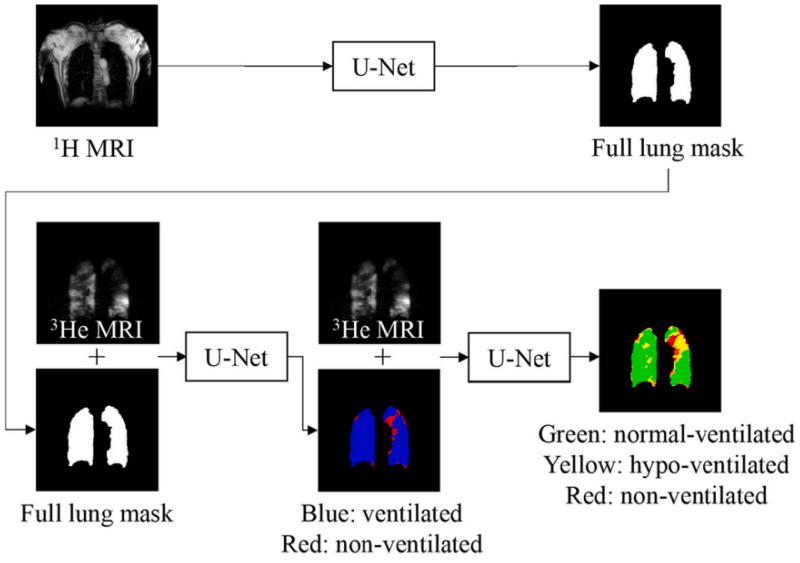
A traditional U-Net was used to segment the full lung mask from the ^1^H MRI. For the segmentation of ^3^He, a two-layer cascaded U-Net was used. Initially, the ^3^He MRI was divided into ventilated (depicted in blue) and non-ventilated (shown in red) lung regions. Further segmentation was applied to the ventilated regions, categorizing them into normal-ventilated areas (depicted in green) and hypo-ventilated zones (illustrated in yellow) within the lungs [49].

This deep learning framework, with its unique use of separate ^1^H and ^3^He segmentation models and the U-Net architecture, offers a novel approach to lung segmentation. Figure 21 presents the end-to-end deep learning framework proposed in the referenced study.

**Figure 22:**
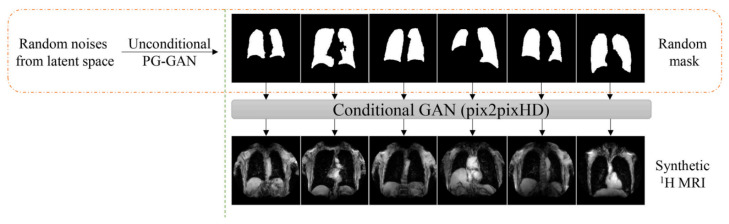
In the GAN-based approach, first, an unconditional GAN, was tasked with generating randomly created complete synthetic lung masks. Second, a conditional GAN played a pivotal role in converting these random masks into synthetic ^1^H MR images [49].

Both conventional and Generative Adversarial Network (GAN)-based data augmentation (GAN-DA) methods were applied to the dataset. Conventional data augmentation (conventional-DA) involved random scaling, translation, rotation, shearing, and flipping. Flipping, while not typically variable in MRI scans, was used to boost model generalization. This technique ensured the model remained unbiased against specific lung patterns that might predominate in left versus right lungs or in apical versus basal and central versus pleural regions. GAN-DA utilized a dual-GAN network approach. The initial GAN [100] was designed to create ^1^H and ^3^He lung masks from random latent noise. Meanwhile, the second GAN, a conditional one using pix2pixHD [101] was developed to generate realistic ^1^H and ^3^He MRI images. The GAN-based DA method is illustrated in Figure 22. 

Table 4 summarizes the four different models.

The deep learning models were developed using Python (version 3.6) and the PyTorch library [102]. Each model was subject to random initialization and trained under specific parameters. The ^1^H lung segmentation model completed 15 epochs using a batch size of 16, whereas the ^3^He ventilated region model was trained with a batch size of 4 for 100 number of epochs. A linear learning rate decay strategy was implemented, which gradually reduced the learning rate from an initial value of 0.0001 to 0. For the ^1^H model, it started from the 5th epoch, and for the ^3^He model, it started from the 50th epoch. The model that demonstrated the best performance on the validation set was then used for test set evaluation.

The loss function was a hybrid, combining cross-entropy (CE) and Dice loss. The CE component compared the probability distributions of predictions and actual labels, while the Generalized Dice Loss (GDL) served as a spatial regularity constraint on the prediction. Both ^1^H and ^3^He models were optimized using the Adam optimizer to minimize the overall loss function. CE, DSC, GDL, and the overall loss function are defined as:

(12) $$CE=-\sum_{w=0}^{W}\;\sum_{h=0}^{H}\;\sum_{c=0}^{C}\;Y(w,h,c)log\;(P(w,h,c))$$

(13) $$DSC=\frac{1}{W\times H\times C}\sum_{w=0}^{W}\;\sum_{h=0}^{H}\;\sum_{c=0}^{C}\;\frac{2\times Y(w,h,c)Y^{\prime }(w,h,c)}{Y(w,h,c)+Y^{\prime }(w,h,c)}$$

(14) $$GDL=1-\frac{2}{W\times H\times C}\sum_{w=0}^{W}\;\sum_{h=0}^{H}\;\sum_{c=0}^{c}\;\frac{{Weight}_{c}\times Y(w,h,c)\times P(w,h,c)}{{Weight}_{c}\times Y(w,h,c)+P(w,h,c)}$$

(15) L=CE+GDL

To verify that the models were not overfitting, four-fold cross-validation was conducted. The dataset was randomly divided into four groups, each serving as a test dataset, with the remaining three groups used for training. A summary of ^1^H lung segmentation and ^3^He ventilated region segmentation parameters are provided in Table 5.

#### 4.4.3. Results

The evaluation of segmentation models involved DSC and Mean Surface Distance (MSD). These metrics were applied to the entire 3D volume of the full lungs. For comparing various DA strategies, both DSC and MSD were considered. Additionally, the effectiveness of the cascaded U-Net model was contrasted with that of the standard U-Net in segmenting ^3^He ventilation defects. Statistical analyses, such as the Wilcoxon signed-rank test, were used to evaluate differences in DSC and MSD outcomes between GAN-DA and traditional DA techniques for the ^1^H segmentation model. Similar methods were used to assess differences between cascaded and standard U-Net models for the ^3^He segmentation model. The Wilcoxon signed-rank test was also applied to contrast actual ground truth values with predicted values for the overall lung volume determined with the ^1^H model. Additionally, this test was used to compare the volumes of non-ventilated, hypo-ventilated, and normally ventilated regions as identified with the ^3^He model.

In relevant instances, the Bonferroni–Holm correction method was used. To assess the differences in model performance between participants with and without COPD, the Mann–Whitney U test was utilized. The Kruskal–Wallis H Test was applied to compare model performances across a four-fold cross-validation. All statistical procedures were conducted using SAS 9.4, and significance was determined using two-tailed tests with a significance level set at α = 0.05. As for the ^1^H MRI segmentation, the DSC showed improvement with the combined DA model (0.965 ± 0.010) and the conventional-DA model (0.961 ± 0.011) when compared with the non-DA model (0.955 ± 0.012). However, the GAN-DA model (0.959 ± 0.010) did not show a significant improvement. 

MSD was lower in the conventional-DA model, averaging 0.594 ± 0.137 mm, in contrast to the non-DA model, which had a higher MSD of 1.055 ± 0.953 mm. Models utilizing a combined-DA approach further decreased the surface distance to an average of 0.657 ± 0.254 mm compared with the non-DA model. However, the MSD for the GAN-DA model, at 0.928 ± 0.690 mm, did not show a significant difference from the non-DA model’s performance. No notable difference was observed between the total lung volume as predicted with deep learning and the actual ground truth total lung volume (3.727 ± 1.075 L compared to 3.721 ± 1.109 L). These findings suggest that both the combined-DA and conventional-DA models enhanced segmentation performance relative to the non-DA model, while the GAN-DA model did not demonstrate a significant enhancement. For the ^3^He segmentation, GAN-DA and combined-DA did not enhance the segmentation of ^3^He MRI; hence, only conventional-DA was applied in ^3^He model training. The cascaded U-Net model achieved DSCs of 0.840 ± 0.057, 0.715 ± 0.175, and 0.883 ± 0.060 for non-ventilated, hypo-ventilated, and normal-ventilated regions, respectively. 

The cascaded U-Net model outperformed the traditional U-Net model in terms of DSC and MSD for non-ventilated regions. There was no significant difference in DSCs and MSDs between COPD and non-COPD participants.

The volumes for non-ventilated, hypo-ventilated, and normally ventilated regions, as predicted with deep learning, did not significantly deviate from their corresponding ground truth volumes. Additionally, segmentation using the cascaded U-Net demonstrated greater alignment with the ground truth compared with segmentation performed with the traditional U-Net. In a four-fold cross-validation, no overfitting was observed in either the traditional U-Net or the cascaded U-Net. A comparison of the traditional U-Net and cascaded U-Net is provided in Table 6.

Based on the literature reviewed, the cascaded U-Net framework streamlined the ventilation segmentation workflow, yielding outputs that align with semi-automated benchmarks. 

**Figure 23:**
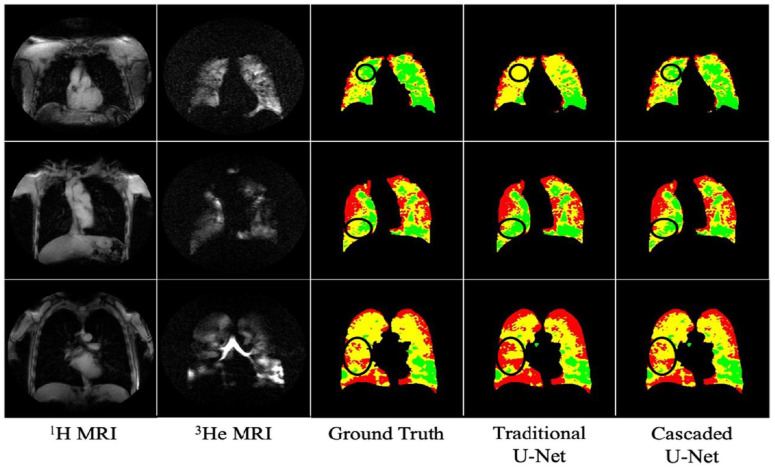
The color-coded regions represent non-ventilated (red), hypo-ventilated (yellow), and normal-ventilated (green) lung areas. The encircled sections emphasize areas where the cascaded U-Net segmentation aligns more closely with the ground truth compared with the traditional U-Net [49].

Both conventional and combined data augmentation techniques markedly enhanced the segmentation of full lung masks from baseline levels. In a similar vein, the cascaded U-Net notably boosted the accuracy of segmenting non-ventilated areas compared with the traditional U-Net. This advanced cascaded U-Net used a multi-layered, hierarchical approach to tackle multi-class segmentation, leveraging the stratified nature of lung ventilation. Table 7 presents the results of ventilation defect segmentation using the cascaded U-Net for participants with and without COPD. In conclusion, the end-to-end deep learning model in the referenced study significantly reduced analysis time compared with previous clustering methods, providing accurate full lung segmentation from ^1^H MRI and multi-categorical ventilation segmentation from ^3^He MRI with a multi-channel scheme. Figure 23 showcases a comparative analysis of the results of ventilation segmentation, contrasting the performance between the traditional U-Net and the cascaded U-Net.

### 4.5. A Dual-Channel Deep Learning Approach for Lung Cavity Estimation from Hyperpolarized Gas and Proton MRI

The paper titled “A Dual-Channel Deep Learning Approach for Lung Cavity Estimation From Hyperpolarized Gas and Proton MRI” [103] presents a deep learning method for estimating lung cavity volumes from MRI images. The study utilizes a dual-channel approach that integrates hyperpolarized gas and proton MRI to generate lung cavity estimations (LCEs) without manual intervention. This method is evaluated against manual estimations, with the goal of producing biomarkers such as the VDP automatically.

The paper discusses various metrics for quantitative and clinical evaluation, including the Dice similarity coefficient, average boundary Hausdorff distance, and a relative error metric. These metrics assess the overlap and conformity of boundaries between ground truth and predicted segmentation, as well as the accuracy of the LCE volumes.

The results indicate that the dual-channel deep learning-generated LCEs are comparable to manual estimations, suggesting that this method could be used to produce lung function biomarkers efficiently.

#### 4.5.1. Materials and Methods

The study obtained ethical approval and informed consent from all participants, including retrospective data usage. It included MRI scans from 26 healthy individuals and 289 patients with lung conditions. The scans were acquired using a 1.5T MRI scanner with specific settings for both hyperpolarized ^129^Xe and proton ^1^H MRI to capture lung images at different capacities.

For image quality, artifacts were identified by experienced observers, and signal-to-noise ratios were calculated to assess noise levels. Lung cavity estimations (LCEs) were initially segmented using a semi-automatic method that combined spatial fuzzy c-means clustering with manual editing by several observers with varying levels of experience.

The study’s MRI data were split into training and testing sets, with 15% of the scans designated for testing. To avoid bias, each participant was represented by only one scan in the dataset, and any additional scans were excluded. The training set consisted of 422 scans from 257 individuals, while the testing set included 58 scans from 58 different participants. The testing set was randomly selected but ensured representation from each health and disease category. Demographically, the training set had a median age of 41 years and a slightly higher proportion of females, while the testing set had a median age of 53 years with an almost equal gender distribution.

#### 4.5.2. Deep Learning-Based Segmentation Architecture

The study evaluated three deep learning methods for generating LCEs using different input channels for each neural network:

- Ventilation-only method using ^129^Xe-MRI images.
- Structural-only method using ^1^H-MRI images.
- Dual-input method combining ^129^Xe-MRI and ^1^H-MRI images.

These methods utilized a 3D version of the UNet [84] architecture, optimized for memory efficiency and allowing for 30 feature channels. The networks were trained with specific parameters to minimize overfitting and were trained for 300 epochs over approximately 8 days on a powerful GPU.

Data augmentation techniques involving random rotation and scaling were applied to the MRI scans before they were input into the network to enhance the robustness of the model without increasing the dataset size.

The DL-generated LCEs were quantitatively evaluated using the DSC for overlap accuracy, the average boundary Hausdorff distance (HD) for boundary conformity, and a relative error metric (XOR) to estimate the manual editing time required for corrections.

Clinically, the study assessed the accuracy of the DL-generated LCE volumes and calculated the VDP using a previously developed nn-UNet [90] for ventilated lung segmentation. This allowed for the comparison of DL-derived VDPs with those derived from manual segmentations. Ventilated volumes were determined using a binning method, with ventilation defects identified as values below 33% of the mean signal intensity in the lung cavity. 

#### 4.5.3. Results

The study’s results showed that the dual-input deep learning method, which uses both ^129^Xe-MRI and ^1^H-MRI images, produced the most accurate LCEs compared with manual segmentations. This method achieved high median scores across various metrics, including the DSC, HD, and XOR.

Clinically, the dual-input DL method demonstrated a strong correlation with manual LCEs, with minimal bias in lung volume measurements. This method’s VDP values also showed no significant difference from those obtained manually, indicating its potential reliability in clinical assessments.

The study also assessed the impact of image artifacts on the quality of DL-generated LCEs. It was found that artifacts in ^129^Xe-MRI scans significantly affected the VDP differences between manual and DL methods, whereas artifacts in ^1^H-MRI scans did not. Additionally, no significant correlation was observed between the SNR and the differences in VDP, suggesting that the SNR did not notably influence the DL segmentation performance.

Three failure cases were highlighted where the DL-generated VDPs deviated from the manual assessments beyond acceptable limits. These included errors due to gas motion artifacts, zipper artifacts, and excessive noise in MRI scans, which led to inaccuracies in the DL-generated LCEs. 

In conclusion, the authors developed a dual-channel CNN framework for LCE, capitalizing on the complementary strengths of both ^1^H-MRI and ^129^Xe-MRI scans. This approach yielded superior performance over single-channel methods, effectively integrating functional and structural imaging data to enhance disease diagnosis in both adult and pediatric populations. The researchers also incorporated an automated deep learning-based technique for ventilated lung segmentation from hyperpolarized gas MRI scans, which facilitated the accurate computation of a clinical biomarker, the VDP. Despite its robustness against noise, the method’s performance declined in the presence of artifacts in ^129^Xe-MRI scans.

A qualitative evaluation highlighted the limitations of using either ventilation or structural data alone, with the dual-input method successfully addressing misalignment issues between the MRI modalities. This was attributed to the method providing adequate contextual information for accurate LCE representation. The application of nn-UNet within the dual-channel approach also allowed for more efficient memory usage, larger batch sizes, and improved handling of volumetric data compared with traditional 2D CNNs. The robustness of the approach was reflected in the limited bias observed in Bland–Altman analysis across a diverse testing set. Furthermore, the deep learning workflow showed the potential for streamlined clinical application, demonstrating the capacity to produce VDPs comparable to manual calculations, significantly reducing the time required for analysis. A quantitative assessment of three methods is reported in Table 8.

Despite the promising results, the study acknowledged limitations, such as the reduced generalizability due to the uniformity of the acquisition protocol and the performance not being tested on different MRI systems or protocols from various centers. Future work suggests strategies to mitigate the impact of imaging artifacts and the possibility of a unified model for both ventilated and structural lung segmentation, although this may currently be limited by the size of the available training datasets.

**Figure 24:**
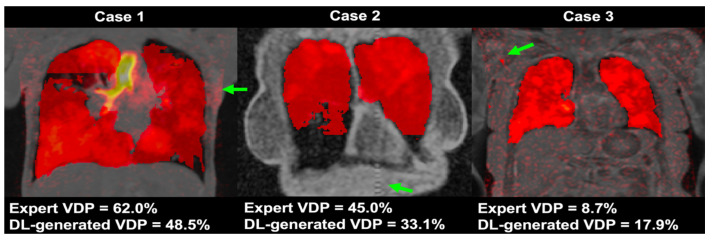
Coronal section examples showing fused ^1^H-MRI, ^129^Xe-MRI, and localized contrast enhancement (LCE) from three distinct cases in the test cohort. These cases were identified as outliers using Bland–Altman analysis, deviating beyond the accepted limits of agreement. The ventilated distribution percentages (VDPs) as assessed by an expert and as generated with deep learning (DL) are displayed for each case. Notable imaging artifacts are marked with green arrows: Case 1 presents a motion artifact within the ^129^Xe-MRI, Case 2 displays a zipper artifact in the ^1^H-MRI, and Case 3 is significantly affected by noise across the ^129^Xe-MRI [103].

Refer to Figure 24 for visual representation of the discrepancies between manual and DL-generated VDP evaluations, along with artifact identification in the MRI slices of the testing set.

## 5. Limitations and Future Work

This paper and the presented discussion provide a comprehensive review of five distinct studies, each exploring the application of deep learning techniques for the segmentation of lung MRI scans, specifically in the context of hyperpolarized gas MRI. The first study focused on the use of 3D CNNs for the segmentation of hyperpolarized gas MRI scans, comparing the performance of different deep learning methods. In the second study, the authors introduced a 3D CNN approach with V-Net architecture to improve lung segmentation in MRI scans. They achieved better results when using both ^3^He and ^129^Xe data. However, the study noted potential limitations, including observer variability in manual segmentations and dataset imbalance. The third study conducted a large-scale investigation of deep learning approaches for ventilated lung segmentation using multi-nuclear hyperpolarized gas MRI. The fourth study developed a deep learning framework for the segmentation of lung MRI scans using a cascaded U-Net model and evaluated its performance on participants with and without COPD. The fifth paper introduced a dual-channel deep learning method for estimating lung cavity volumes from MRI images using hyperpolarized gas and proton MRI data. They used a 3D U-Net architecture, achieving accurate results in lung cavity estimations. However, potential limitations include limited generalizability due to uniform acquisition protocols and decreased accuracy in the presence of artifacts in ^129^Xe-MRI scans.

The collective insights from the five papers suggest that deep learning models, specifically U-Net architectures, are remarkably effective in segmenting lung regions and quantifying ventilation defects from hyperpolarized and proton MRI scans. These studies demonstrate the growing potential of machine learning techniques in enhancing the accuracy and efficiency of pulmonary imaging analysis. Notably, cascaded U-Net models and dual-channel approaches have shown substantial promise in improving the segmentation of non-ventilated and hypo-ventilated lung regions, which is critical for the assessment and management of chronic obstructive pulmonary disease (COPD). The use of data augmentation, including Generative Adversarial Networks (GANs), to enrich training datasets has emerged as a vital strategy to bolster the models’ performance, especially when dealing with limited data scenarios.

Conclusively, these advanced deep learning frameworks offer a leap forward in automating the segmentation and analysis process, providing reliable biomarkers for lung function assessment. The dual-channel method stands out by efficiently combining structural and functional data, enhancing the diagnosis and monitoring of lung conditions in both adult and pediatric cohorts. However, the studies collectively acknowledge the limitations posed by imaging artifacts and the variability in acquisition protocols, highlighting areas for future improvement. As the technology matures and datasets expand, it is anticipated that these AI-driven methodologies will become more robust, paving the way for widespread clinical adoption. Further research is recommended to address the challenges associated with artifacts and to explore the potential of unified models that accommodate diverse imaging systems and protocols. A summarized comparison of the reviewed articles is presented in Table 9.

Despite the significant strides made in these studies, several limitations and potential areas for future research were identified. Firstly, while the models developed and evaluated in these studies showed promising results, they are tailored to the specific datasets used in each study. Future work could focus on creating a more generalized model for segmentation that can handle a wider variety of datasets, increasing its utility in different contexts.

Secondly, the creation of a more accurate and comprehensive dataset that encompasses a similar distribution of different diseases or geographical data could significantly enhance the performance of the models. A more representative dataset would allow the models to learn from a broader range of cases, thereby improving their ability to generalize and handle new, unseen data.

Thirdly, the potential of Vision Transformers (ViTs) in the segmentation of medical images, such as hyperpolarized gas MRI of the lung, is a promising avenue for future research. While CNNs have been the go-to architecture for image segmentation tasks, they primarily focus on local features and spatial hierarchies. This localized focus can sometimes be a limitation when global context is essential for accurate segmentation [104]. Vision Transformers, on the other hand, have the inherent capability to model global interactions across an entire image from the get-go. This is particularly beneficial for medical imaging, where understanding the global context can be crucial for accurate diagnosis [105].

ViTs split the image into patches and treat these patches as tokens, which are then processed by the transformer encoder. The encoder produces a contextualized sequence of tokens that capture both local and global features. This sequence is then upsampled to generate per-pixel class scores for segmentation. Such a mechanism allows ViTs to capture intricate relationships between different parts of an image, thereby potentially improving the segmentation accuracy. Moreover, ViTs have been shown to outperform state-of-the-art CNNs in challenging datasets, achieving higher mean Intersection over Union (IoU) scores [106].

The application of these advanced models to hyperpolarized gas MRI of the lung could potentially yield even more accurate and efficient segmentation results. Given that ViTs can be parallelized easily, they are also more scalable and can be trained on large datasets, which is often a requirement in medical imaging research.

Fourthly, the potential of transfer learning for this task has not been explored in these studies. Transfer learning, which involves the application of knowledge gained from one task to another related task, could potentially enhance the performance of the models. Specifically, the transfer learning of low-resolution data could provide a better foundation for high-resolution data, thereby improving the accuracy and efficiency of the models. 

In conclusion, while the reviewed studies made significant contributions to the field of lung MRI segmentation, there are several potential avenues for future research that could further enhance the performance and utility of deep learning models in this area. The studies demonstrated the power of deep learning in accurately segmenting lung MRI scans, but there is still room for improvement and exploration. These potential avenues for future research highlight the exciting opportunities that lie ahead in the field of lung MRI segmentation using deep learning. The reviewed studies laid a solid foundation, and future work building on this foundation could lead to significant advancements in the field.

## Figures and Tables

**Table 1:** A summary of the U-Net parameters used in the referenced study.

Parameter	Proton Model	Ventilation Model
Adam optimization learning rate	0.00001	0.0001
Number of epochs	150	150
Training/validation data split	80/20	80/20
CL ^1^ kernel size	5 × 5 × 5	5 × 5 × 5
CL activation	ReLU	ReLU
CL number of filters	Starts with N = 16, doubles at every layer	Starts with N = 32, doubles at every layer
Dropout layer rate MPL ^2^ size	0.2 2 × 2 × 2	0.2 2 × 2 × 2
MPL stride length	2 × 2 × 2	2 × 2 × 2
U/TCL ^3^ kernel size	5 × 5 × 5	5 × 5 × 5
U/TCL stride length	2 × 2 × 2	2 × 2 × 2
U/TCL activation	ReLU	ReLU

^1^ Convolution layers. ^2^ Max pooling layers. ^3^ Upsampling/transposed convolution layers.

**Table 2:** A summary of the nnU-Net parameters used in the referenced study.

Parameter	Value
Activation function	PReLU
Learning rate (initial training)	1 × 10^−5^
Learning rate (fine-tuning)	0.5 × 10^−5^
Spatial window size	[96, 96, 96]
Batch size	2

**Table 3:** A summary of the performance of each of the five deep learning experimental approaches.

Metric	Description	Formula
DSC (Dice similarity coefficient)	Measures overlap between the ground truth ^1^ and predicted ^2^ segmentations	$DSC=2\frac{\left|PR\;\cap \;GT\right|}{\left|PR|\;+\;|GT\right|}$
Avg HD (average boundary Hausdorff distance)	Average distance between the most distant point of the predicted and ground truth segmentation, and vice versa	Avg HD(PR,GT)=max(d(PR,GT),d(GT,PR))
HD95 (95th percentile Hausdorff distance)	The 95th percentile of the Hausdorff distance was used to remove the impact of outlier voxels	HD(PR,GT)=max(h(PR,GT),h(GT,PR))
XOR (relative error metric)	Evaluates segmentation errors	$XOR=\frac{|PR\;\cap \;GT\prime |\;+\;|\;|PR\prime \;\cap \;GT|\;|}{\left|GT\right|}$

^1^ GT = ground truth. ^2^ PR = predicted.

**Table 4:** A summary of four different DA models.

Model Name	Training Data	Number of Slices (for Both ^1^H MRI and ^3^He MRI)
Non-DA Model	Original coronal slices without any data augmentation	328
Conventional-DA Model	Original coronal slices in addition to 10-fold slices generated with conventional data augmentation	3608
GAN-DA Model	Original coronal slices in addition to 10-fold slices generated with dual-GAN data augmentation	3608
Combined-DA Model	Original coronal slices in addition to five-fold slices generated with both conventional and GAN data augmentation	3608

**Table 5:** Summary of the ^1^H lung segmentation and ^3^He ventilated region segmentation parameters.

Model Training Parameters	^1^H Lung Segmentation	^3^He Ventilated Regions Segmentation
Epochs	15	100
Batch size	16	4
Initial learning rate	0.0001	0.0001
Decay start epoch	5	50
Loss function	CE + Dice Loss	CE + Dice Loss
Optimizer	Adam	Adam

**Table 6:** Evaluation of traditional U-Net vs. cascaded U-Net for ventilation defect segmentation in the test set (sample size: 14).

	Traditional U-Net	Cascaded U-Net	*p*-Value
Non-ventilated DSC	0.818 ± 0.055	0.840 ± 0.057	<0.001
Non-ventilated MSD (mm)	1.432 ± 1.343	1.275 ± 1.396	<0.005
Hypo-ventilated (^3^He) DSC	0.697 ± 0.192	0.715 ± 0.175	0.24
Hypo-ventilated (^3^He) MSD (mm)	1.288 ± 1.357	1.166 ± 1.237	0.30
Normal ventilated (^3^He) DSC	0.875 ± 0.070	0.883 ± 0.060	0.50
Normal ventilated (^3^He) MSD (mm)	1.359 ± 0.845	1.506 ± 1.054	0.76

**Table 7:** Comparative analysis of ventilation defect segmentation using cascaded U-Net in participants with and without COPD in the test set (sample size: 14).

	COPD, n = 8	No COPD, n = 6	*p*-Value
Non-ventilated DSC	0.851 ± 0.056	0.826 ± 0.061	0.41
Non-ventilated MSD (mm)	1.228 ± 1.630	1.338 ± 1.158	0.75
Hypo-ventilated (^3^He) DSC	0.653 ± 0.208	0.799 ± 0.066	0.06
Hypo-ventilated (^3^He) MSD (mm)	1.516 ± 1.561	0.700 ± 0.331	0.66
Normal ventilated (^3^He) DSC	0.861 ± 0.071	0.912 ± 0.024	0.18
Normal ventilated (^3^He) MSD (mm)	1.855 ± 1.158	1.042 ± 0.748	0.08

**Table 8:** Quantitative assessment of ventilation-only, structural-only, and dual-channel DL methods on a testing set of 58 scans. Median values are given.

LCE DL Method	DSC	Average HD (mm)	XOR
Ventilation-only	0.952	2.22	0.095
Structural-only	0.935	4.19	0.132
Dual-channel	0.967	1.68	0.066

**Table 9:** Overview of key studies reviewed in this paper. The table compares various papers in terms of their objectives, applied methods, data used for analysis, principal findings, and recognized limitations.

Paper Title	Objective	Methods	Data	Key Findings	Limitations
Convolutional Neural Networks and template-based data augmentation for functional lung Image quantification [48]	To explore the potential of CNNs for functional lung imaging and propose a data augmentation strategy for addressing the need for large training datasets.	U-Net architecture for image segmentation, multilabel Dice coefficient loss function, and a novel template-based data augmentation method.	A total of 205 proton MR images and 73 ventilation MR images with masks, from ^3^He and ^129^Xe acquisitions.	Achieved high Dice overlap scores for lung segmentation, with a significant reduction in processing time compared with conventional methods.	Limited by GPU memory capacity, necessitating the use of 2D over 3D U-Net models and processing time-intensive training.
3D deep convolutional neural network-based ventilated lung segmentation using multi-nuclear hyperpolarized gas MRI [94]	To enhance the segmentation of ventilated lung regions in MRI scans using a 3D deep convolutional neural network.	Utilized V-Net architecture with PReLu activation functions and binary cross-entropy loss, optimized with the Adam optimizer.	A total of 743 volumetric hyperpolarized gas MRI scans from 326 subjects, including both ^3^He and ^129^Xe gases.	Presented a novel approach that improved segmentation accuracy over traditional methods, with best results when training on both ^3^He and ^129^Xe data.	Potential observer variability in manual segmentations and dataset imbalance were noted as limitations.
Large-scale investigation of deep learning approaches for ventilated lung segmentation using multi-nuclear hyperpolarized gas MRI [47]	To identify the most effective 3D CNN architecture, loss function, and pre-processing techniques for the segmentation of ventilated lungs in hyperpolarized gas MRI scans.	Use of nn-UNet for 3D volumetric convolutions and spatially adaptive denoising, along with comprehensive experiments across five deep learning methods.	A total of 759 volumetric hyperpolarized gas MRI scans from 341 subjects, including healthy individuals and patients with a range of pulmonary conditions.	The combined training method using both ^3^He and ^129^Xe gases was the best-performing method, producing accurate segmentations and demonstrating unbiased performance irrespective of the noble gas used. CNNs proved superior to traditional methods in all evaluation metrics.	The study calls for further validation of DL-based methods using ventilated lung volume and ventilation defect percentage for clinical adoption.
Quantification of lung ventilation defects on hyperpolarized MRI: The Multi-Ethnic Study of Atherosclerosis (MESA) COPD study [49]	To develop and validate a deep learning framework for segmenting lung MRI scans into non-ventilated, hypo-ventilated, and normal-ventilated regions.	The study uses a cascaded U-Net model and conventional and GAN-based data augmentation to improve the robustness of the segmentation model.	Utilized data from the MESA COPD study involving 56 participants with significant smoking histories, resulting in 544 slices each of 1H and ^3^He pulmonary MRI.	The cascaded U-Net model showed high accuracy and consistency with semi-automatic reference standards for both COPD and non-COPD participants. Conventional data augmentation improved the segmentation performance compared with the non-augmented data model.	The study mentions manual ROI selection for threshold determination and the potential for error propagation from original to augmented data, and it stresses the need for a consensus on lung ventilation segmentation methods.
A dual-channel deep learning approach for lung cavity estimation from hyperpolarized gas and proton MRI [103]	To develop a deep learning approach for estimating lung cavity volumes from MRI images using a dual-channel method that integrates hyperpolarized gas and proton MRI.	The study utilized a 3D U-Net architecture with 30 feature channels and applied data augmentation for robust training. It compared three deep learning methods using different input channels to produce lung cavity estimations (LCEs).	Included MRI scans from 26 healthy individuals and 289 patients with lung conditions, obtained using a 1.5T MRI scanner.	The dual-input method, combining hyperpolarized ^129^Xe -MRI and proton 1H-MRI images, provided the most accurate LCEs compared with manual segmentations. It showed a strong correlation with manual estimations and consistent VDP values.	The study’s generalizability might be limited due to the uniformity of the acquisition protocol, and its performance has not been tested on different MRI systems or protocols from various centers. The method’s accuracy decreases in the presence of artifacts in ^129^Xe -MRI scans.
